# Polysorbate 80 surface modified SLNs of formoterol suppress SNCA gene and mitochondrial oxidative stress in mice model of Parkinson’s disease

**DOI:** 10.1038/s41598-023-46511-3

**Published:** 2023-11-15

**Authors:** Piyong Sola, Kusuma Kumari Garikapati, Praveen Thaggikuppe Krishnamurthy, Mamta Kumari

**Affiliations:** Department of Pharmacology, JSS College of Pharmacy, JSS Academy of Higher Education & Research, Ooty, 643001 Tamil Nadu India

**Keywords:** Biological techniques, Cell biology, Drug discovery, Molecular biology, Neuroscience, Diseases, Medical research, Molecular medicine, Neurology, Nanoscience and technology

## Abstract

The present study hypothesises that the selective brain β2 receptor activation through β2-adrenoreceptor agonist (β2ARA), Formoterol (FMT), suppresses SNCA gene expression, a pathological hallmark of Parkinson’s disease (PD) in brain. Further, it is also hypothesized that brain targeted delivery of Formoterol via polysorbate-80 surface modified solid lipid nanoparticles of Formoterol (FMT-SLNs-PS80) can improve its stability, therapeutic efficacy and avoid/reduce peripheral off-target side effects. FMT-SLNs-PS80 was prepared by solvent injection method, the formulation was optimized by using Box–Behnken design and characterized by measuring drug content, entrapment efficacy, particle size, zeta potentials and poly dispersibility. The FMT-SLNs-PS80, significantly decreases the SNCA expression, mitochondrial membrane damage and rotenone induced changes in oxidative (SOD, CAT, GSH and ROS) stress markers in SH-SY5Y cell lines. The ex vivo permeation study of the formulation using everted chicken ileum exhibited a steady state flux. The pharmacokinetic and tissue distribution studies of the formulation in rats showed a significant improvement in the kinetic parameters when compared to naïve FMT, further the formulation also improved the brain bioavailability of FMT. The anti-Parkinson’s efficacy studies of the formulation in mice showed a significant neuroprotection against rotenone-induced changes in behavioural and biochemical parameters. Further, the histopathological analysis of mice brain confirms a significant neuroprotective benefit. The present study successfully establishes the brain targeted delivery and anti-Parkinson’s therapeutic efficacy of FMT-SLNs-PS80.

## Introduction

Parkinson's disease (PD) is a chronic progressive neurodegenerative disorder characterized by dopaminergic (DA) neurons degeneration in the substantia nigra of the midbrain and by the appearance of Lewy bodies, which are intracellular inclusions of aggregated alpha-synuclein (SNCA)^1,2^. PD is reported to be the fastest growing brain disorder, the most prevalent movement disorder and the second most prevalent neurodegenerative disease affecting the world. As per the Global Burden of Disease Study 2019, PD accounts as the second largest number of dementia cases, totalling 1.71 million cases. Globally, an estimated 9.4 million people are living with PD in 2020^3^. This rise in prevalence depicts the societal burden which necessitates the measures to find novel, safe, and efficacious agents for the better management of PD.

The current treatment strategy for PD aims at increasing dopamine levels. The medications such as Monoamine oxidase B (MAO-B) inhibitors, catechol o-methyltransferase (COMT) inhibitors, Levodopa, dopamine agonist and dopamine reuptake inhibitors are focused on the motor complications and therefore provide only symptomatic relief^4^. Neurodegeneration is considered to be one of the major risk factors in PD and is responsible for the development of life-threatening complications such as depression, dysphagia, or difficulty in swallowing, cognitive impairment, sleep disorder, etc^5^. Unfortunately, none of the anti-parkinsonian therapies, alone or in combination have the ability to halt disease progression on a long-term basis. It is, therefore, important to halt disease progression with neuroprotective agents to effectively manage this disease.

β2-adrenoreceptor (β2AR) agonists are reported to have neuroprotective benefits in PD. β2-AR agonist, clenbuterol, is found to regulate the transport of abundant neutral amino acids across the blood–brain barrier (BBB)^6^. Studies show that in monkeys, the transport of levodopa to the brain is reduced in severe PD and which could be increased by prior administration of the β2-AR agonist, isoproterenol^7^. Mittal et al. reported that β2AR is linked to the transcription of SNCA, which increases the risk of PD, thus possibly a novel target for the development of anti-Parkinson therapy. Besides, β2AR agonists are reported to promote dopaminergic neuron health by reducing mitochondrial oxidative stress^8^. The above scientific evidence therefore makes a strong case for these drugs as potential anti-Parkinson agents. β2AR agonists are reported to show peripheral side effects such as tachycardia, palpitation, pulmonary edema, myocardial ischemia, and cardiac arrhythmia due to activation of βAR in the peripheral tissues. One of the criteria, therefore, for repurposing β2AR agonists as anti-Parkinson agents is to avoid/minimize peripheral side effects. One of the strategies therefore is to deliver β2AR agonists specifically to brain tissue. In the present study, we prepared a selected β2AR agonist, Formoterol (FMT) as Solid Lipid Nanoparticles (SLNs) surface modified with polysorbate-80 (PS80) (FMT-SLNs-PS80) and evaluated its brain specific delivery and anti-Parkinson’s efficacy.

## Results

### Formulation and optimization of FMT-SLNs-PS80 for brain targeted delivery

The compatibility studies of stearylamine (SA) and Formoterol (FMT, base form) were carried out using differential scanning calorimetry (DSC) and Fourier-transform infrared spectroscopy (FT-IR). No peak shifting was observed which confirms that they are compatible (Supplementary information Figs. S4 and S5).

The physicochemical characteristics such as particle size (PS), zeta potential (ZP), and Polydispersity index (PDI) of trial batch FMT-SLNs-PS80 as well as blank SLNs are summarized in Table 1 and Supplementary information Fig. S6.

**Table 1:** PS, ZP and PDI of trial batch FMT-SLNs-PS80 and blank SLNs.

Formulation	PS (nm)	ZP (mV)	PDI (%)
FMT-SLNs-PS80	154.81 ± 7.92	22.11 ± 3.18	0.264 ± 0.01
Blank SLNs	122.01 ± 0.30	17.86 ± 1.81	0.261 ± .02

The formulation was optimised by Box–Behnken design (BBD). 15 experimental runs were carried out and the response data is presented in Supplementary information Table S4. The effects of independent variables on PS (nm) and ZP (mV) were detected by the response analysis of variance (ANOVA) (Supplementary information S.1.2.1, Table S5, S.1.2.2 and Table S6), and their main effects plots are represented in Supplementary information Figs. S8, S9.

On the basis of desirability (near to 1), an optimal formulation was chosen. The software estimated that a surfactant concentration of 4.29% w/v, sonication amplitude of 76.26%, and a sonication time of 8.72 min would be necessary to create FMT-SLNs-PS80 with a PS of 154.88 nm and a ZP of 24.26 mV (Supplementary information Fig. S7). A new batch of FMT-SLNs-PS80 formulation was prepared and the experimental findings show FMT-SLNs-PS80 with PS of 151.28 ± 0.33 nm and ZP of 22.11 ± 3.18 mV, respectively, with prediction errors of 3.61 ± 0.33 and 2.16 ± 3.18 (Table 2).

**Table 2:** PS and ZP of predicted and experimental batches of optimized FMT-SLNs-PS80.

	Predicted value	Experimental value	Prediction error
PS (nm)	154.88	151.28 ± 0.33	3.61 ± 0.33
ZP (mV)	24.26	22.11 ± 3.18	2.16 ± 3.18

The values are mean ± SD (n = 3).

Further, the overall physicochemical parameter of the optimized batch of FMT-SLNs-PS80 is given in Table 3. The SEM and TEM studies revealed a spherical shape of the optimized batch of FMT-SLNs-PS80 (Supplementary information Fig. S10).

**Table 3:** Physicochemical parameters of optimized batch of FMT-SLNs-PS80.

Parameter	Value
Particle size (PS) (nm)	151.28 ± 0.33
Zeta potential (ZP) (mV)	22.11 ± 3.18
Polydispersity index (PDI)	0.143 ± 0.11
Drug content (%)	98 ± 0.03
Drug loading (DL) (%)	8.97 ± 0.01
Entrapment efficiency (EE) (%)	89.70 ± 0.12

The values are mean ± SD (n = 3).

**Figure 1:**
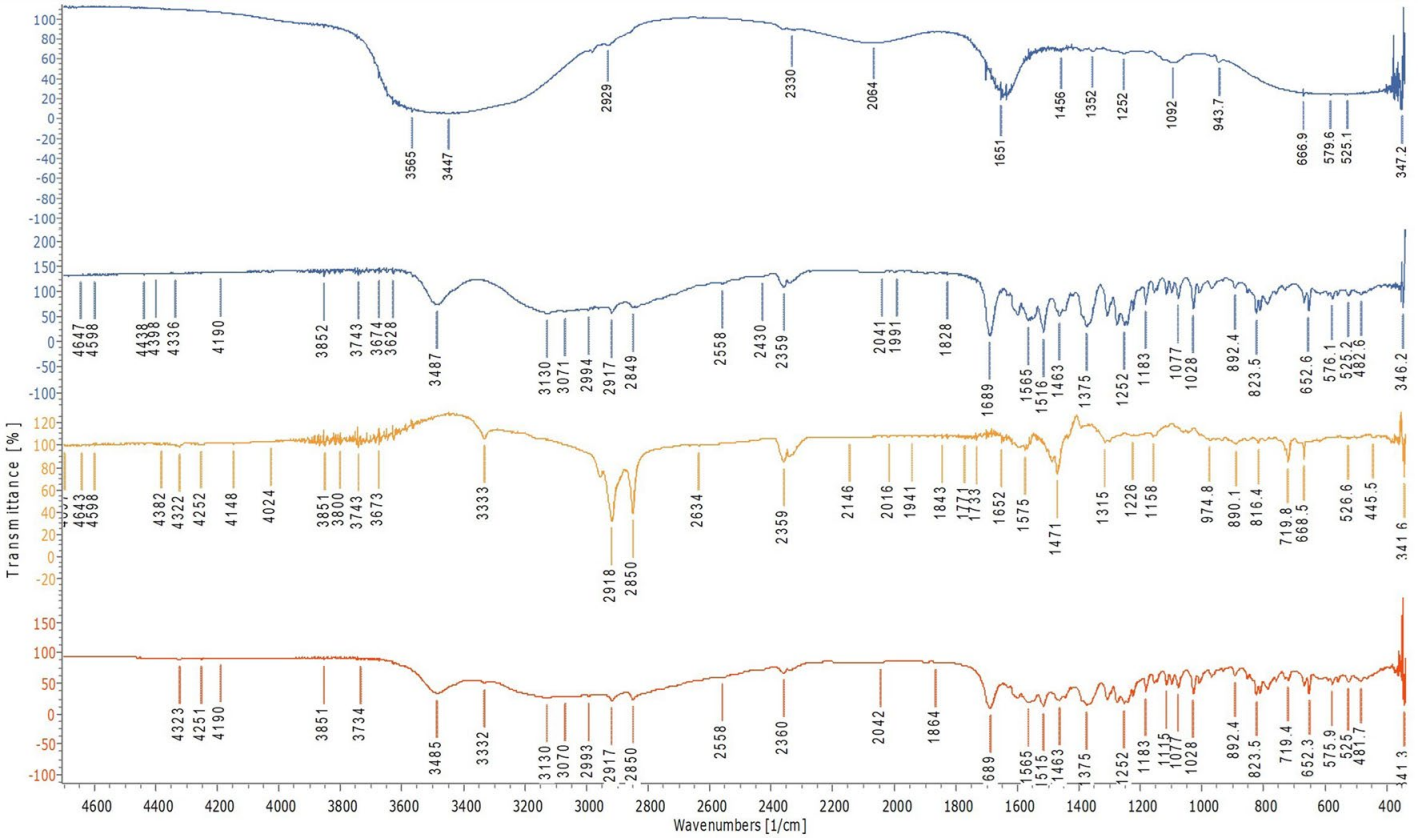
FT-IR spectra of FMT-SLNs-PS80, Naïve FMT, Blank SLNs and physical mixture (FMT + SA).

**Figure 2:**
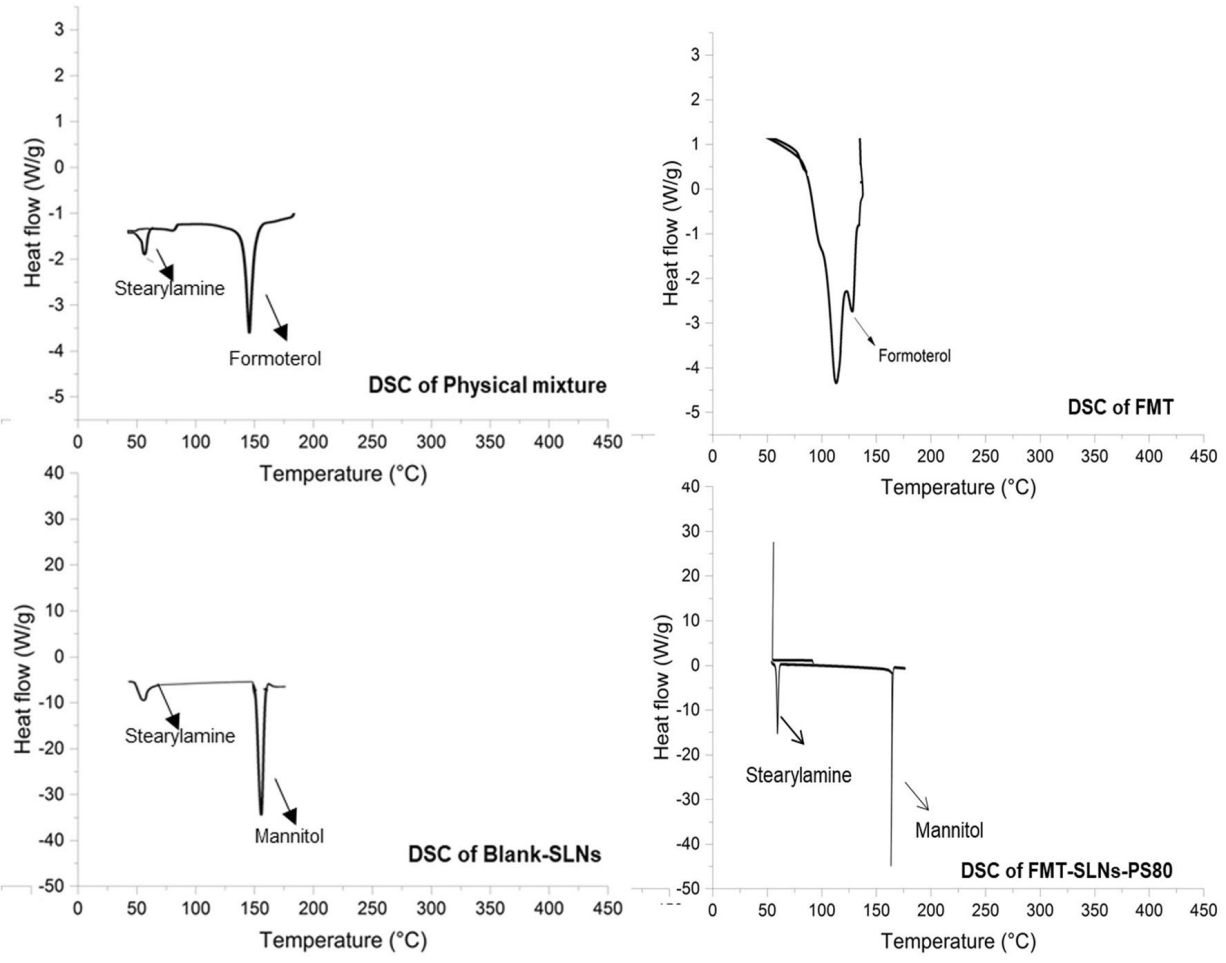
DSC of physical mixture (FMT + SA), FMT, Blank SLNs and FMT-SLNs-PS80.

To confirm the entrapment of drug in the formulation, FT-IR was performed and compared with spectra of FMT (Fig. 1). The principal functional group peaks of the FMT were absent in the FT-IR spectrum of the formulation indicating the entrapment of the drug. Also, the DSC result of the Blank SLNs given in Fig. 2 shows the endometric peak of mannitol and stearylamine. The DSC of FMT-SLNs-PS80 showed the endometric peak of mannitol at 151.54 °C, which was added as a cryoprotectant during the lyophilization of the SLNs. The FMT peak was absent in the formulation indicating the conversion of the drug into the amorphous form in the SLNs (Fig. 2).

**Figure 3:**
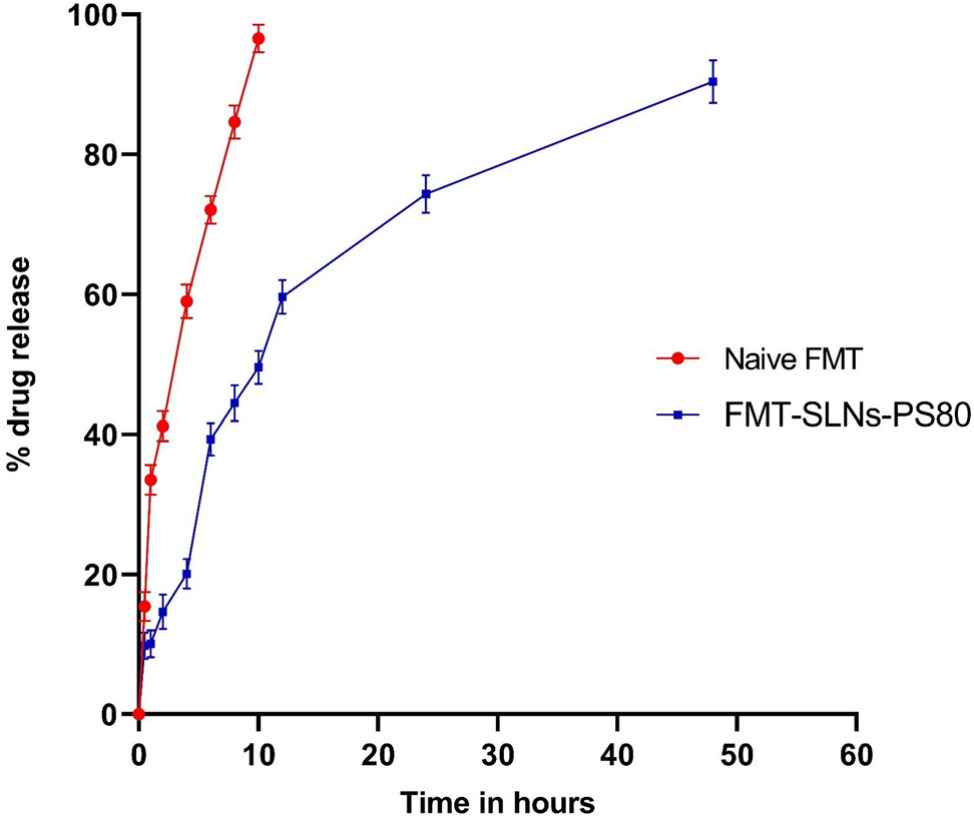
In vitro drug release plot for Naïve FMT and FMT-SLNs-PS80.

The in vitro drug release study result is summarized in Fig. 3. The release of naive FMT from the solution was found to be quicker than FMT-SLNs-PS80. The naïve FMT was almost totally (96.54 ± 1.91%) released from the solution at the 10th hour. FMT-SLNs-PS80, on the other hand, displayed a biphasic release pattern with an initial burst release lasting till the 6th hour and then a continuous release up to the 48th hour (90.42 ± 3.07%) (Fig. 3).

Further, the data obtained was fitted into various release kinetic models to know the release kinetics of optimised FMT-SLNs-PS80 (Supplementary information Figs. S11–S15). The R^2^ value (Table 4) depicts that the release of FMT from FMT-SLNs-PS80 follows first-order release kinetics. The n-value reveals the release mechanism of drug. Our result yield n-value of 0.605 which shows that formulation, FMT-SLNs-PS80 obeys non-Fickian diffusion mechanism.

**Table 4:** In vitro release kinetics data of optimised FMT-SLNs-PS80.

Release kinetics	R^2^
Zero order	0.7934
Higuchi	0.9572
Korsmeyer–Peppas	0.9220 n = 0.605
First order	0.9777
Hixson Crowell	0.9316

**Figure 4:**
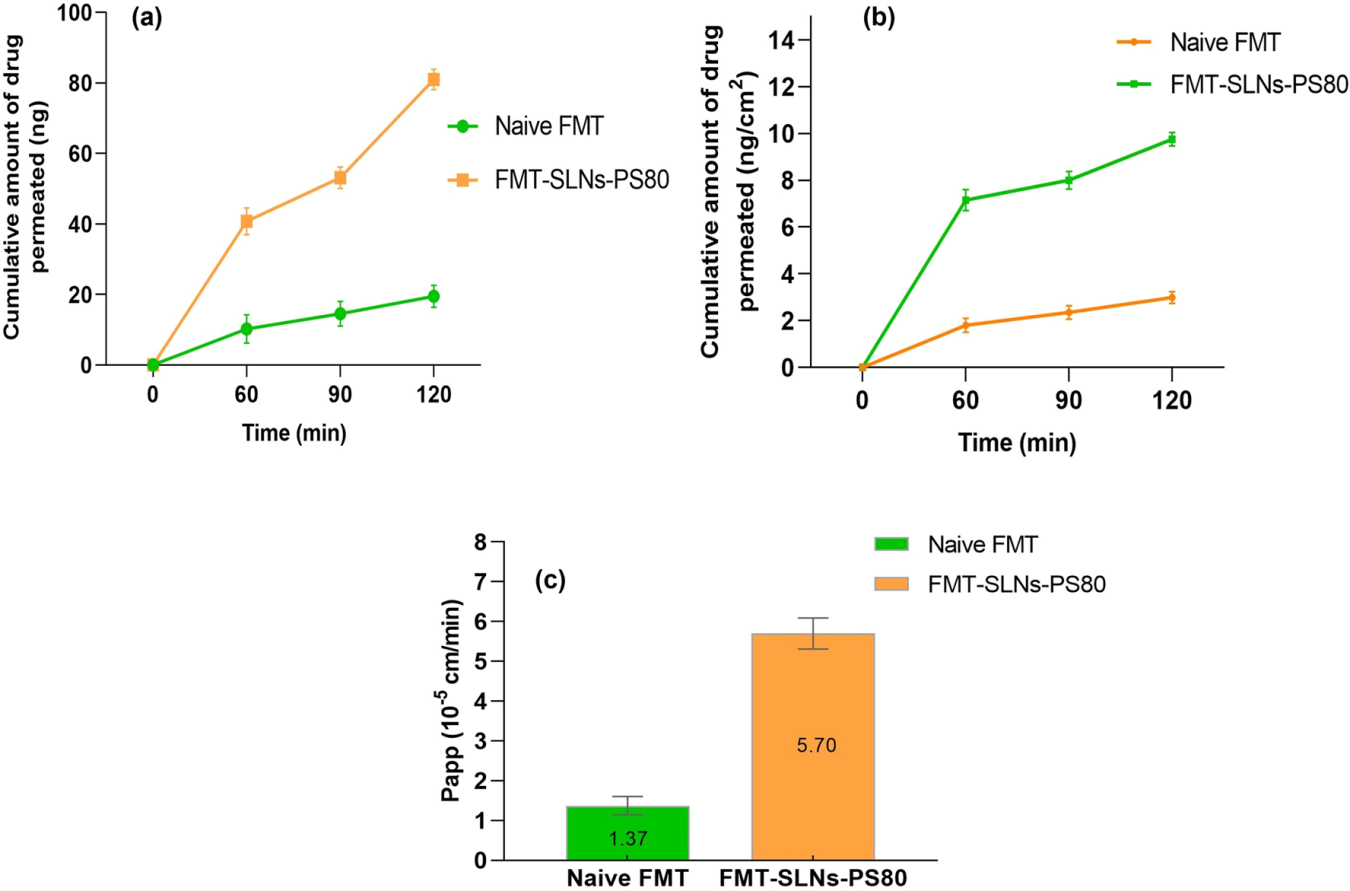
Ex vivo permeation studies: (**a**) Cumulative amount of drug permeated (ng) versus time (min), (**b**) Cumulative drug transported (ng/cm^2^) versus time (min), and (**c**) P_app_ for Naïve FMT and FMT-SLNs-PS80 formulation.

In order to know the permeability of FMT-SLNs-PS80, ex vivo permeation study was performed using everted sac model. The result reveals that the formulation, FMT-SLNs-PS80 has better permeation than naïve FMT solution (Fig. 4). The permeation flux (F) was calculated by plotting the cumulative amount of FMT permeated (ng) versus time (min) (Fig. 4a). Furthermore, the amount of FMT transported through the intestinal sac from FMT-SLNs-PS80 was plotted against time (Fig. 4b), which reveals that larger amount of FMT was transported through the intestinal sac from FMT-SLNs-PS80 than from naive FMT solution. The apparent permeability coefficient (P_app_) was measured for the transport of naïve FMT solution and was compared with FMT-SLNs-PS80 (Fig. 4c), where the result showed that FMT-SLNs-PS80 has higher P_app_ as compared to naïve FMT solution. The increased permeability of the FMT-SLNs-PS80 may be due to SA which was used as lipid, PS80 as surfactant and surface modifying agent in the formulation. PS80 is reported to inhibit the intestinal P-gp thus increasing the permeability and may aid in the absorption of the FMT as they are capable of modifying membrane permeability and hence improving the absorption across the gut^9^.

### In vitro neuroprotective activity of FMT-SLNs-PS80

**Figure 5:**
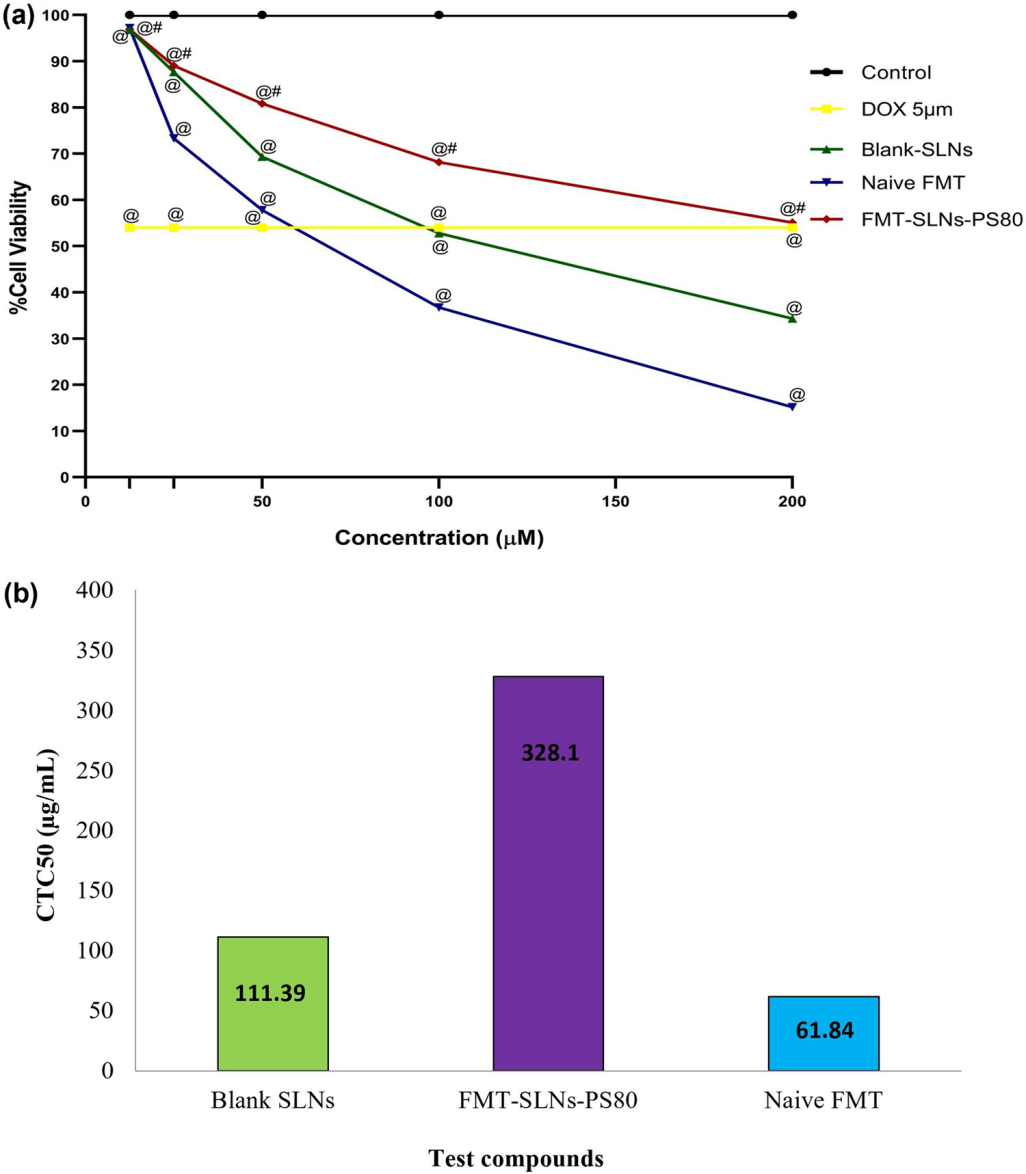
(**a**) Percentage cell viability of SH-SY5Y cell lines. The data represent mean ± SD (n = 3); ^@^p < 0.05 versus Control; ^#^p < 0.05 versus Naïve FMT. (**b**) Comparative CTC_50_ values of Blank SLNs, FMT-SLNs-PS80 and Naïve FMT against SH-SY5Y cells.

MTT assay was performed to assess the viability of SH-SY5Y cell lines. The results were presented in Fig. 5. Upon 24 h incubation with 12.5–200 µg/mL of Naïve FMT and FMT-SLNs-PS80 in the respective groups, the CTC_50_ of Naïve FMT was found to be 61.84 μg/mL whereas, FMT-SLNs-PS80 showed CTC_50_ at 328.1 μg/mL. Hence, indicating the lower cytotoxicity of FMT-SLNs-PS80 in SH-SY5Y cell line as compared to Naïve FMT.

### FMT-SLNs-PS80 suppresses mitochondrial membrane damage

**Figure 6:**
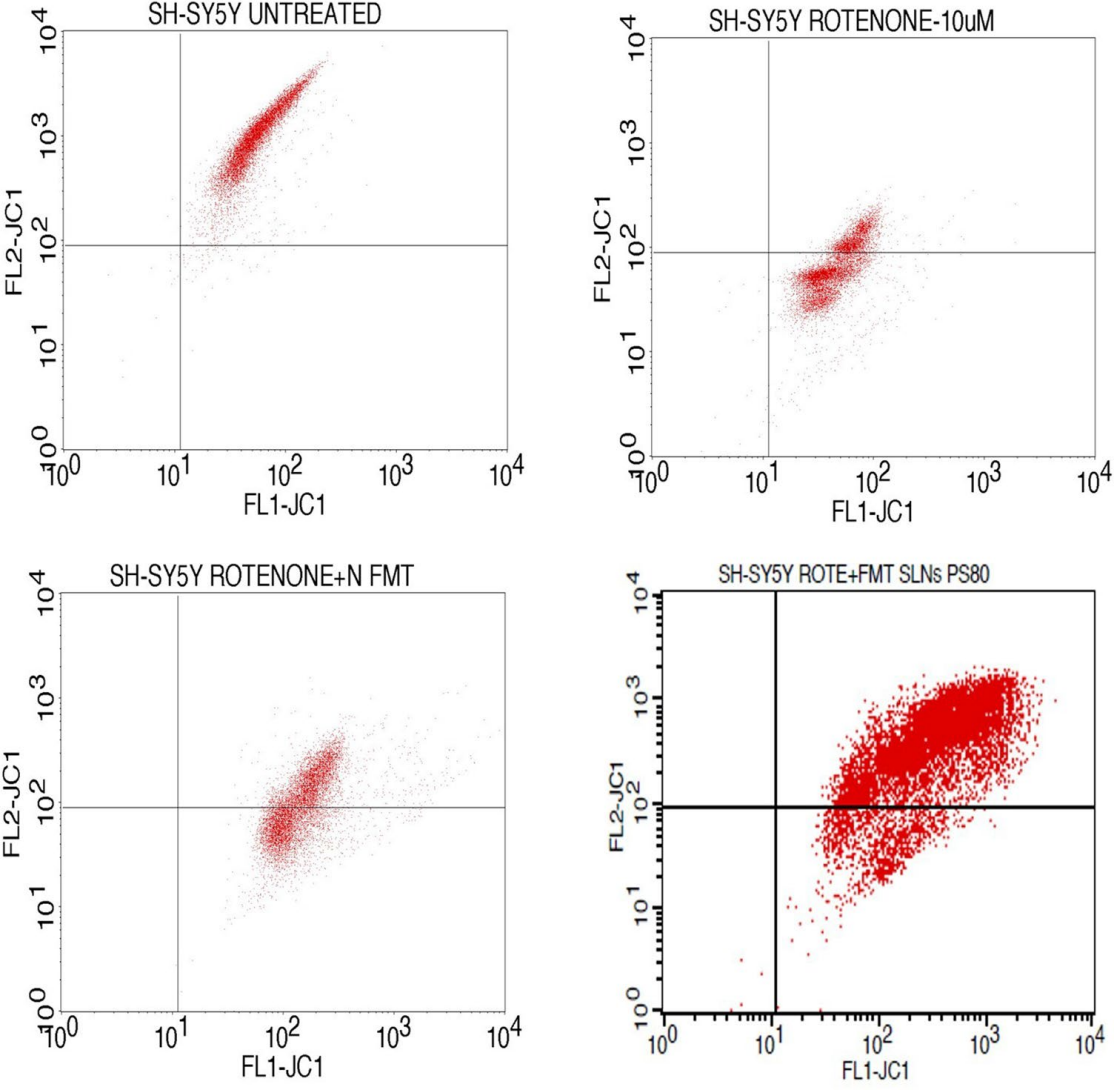
Quadrants showing the expression of JC1 stain in the given Untreated SH-SY5Ycells, Rotenone alone induced cells and Rotenone induced with Naïve-FMT and FMT-SLNs-PS80 treated SH-SY5Y cells against the FL1-JC1 and FL2-JC1.

**Figure 7:**
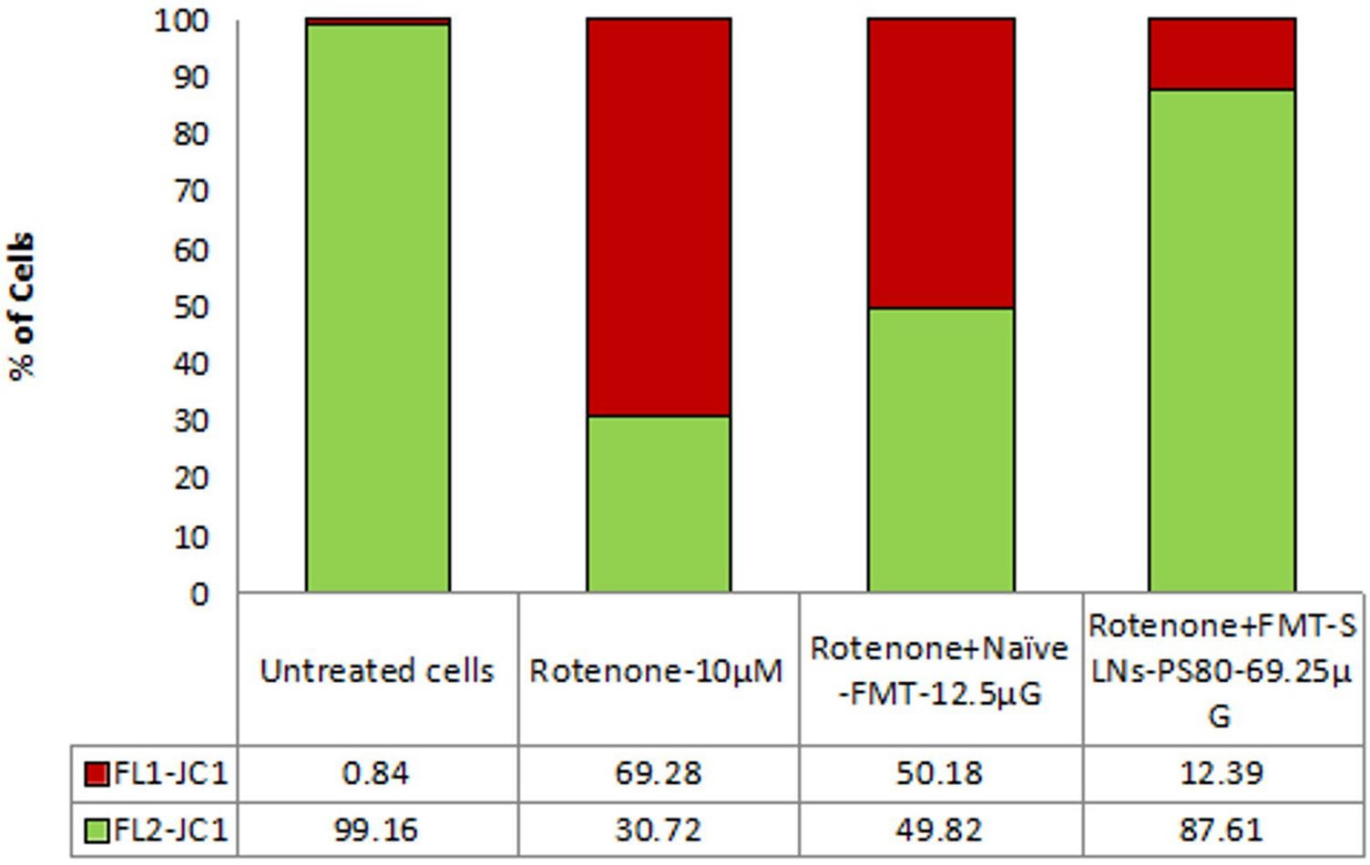
Quadrants showing the expression of JC1 stain in the given Untreated SH-SY5Ycells, Rotenone alone induced cells and Rotenone induced with Naïve-FMT and FMT-SLNs-PS80 treated SH-SY5Y cells against the FL1-JC1 and FL2-JC1.

In this study, effects of Naive FMT and FMT-SLNs-PS80 were evaluated to analyse the mitochondrial membrane potency effect on SH-SY5Y cell lines which are pre induced with rotenone (10 μM). The results (Figs. 6 and 7) reveal that FMT-SLNs-PS80 (69.25 µg/mL) significantly reduce the mitochondrial membrane damage to greater extend as compared to naïve FMT (12.5 µg/mL) against the rotenone induced SH-SY5Y cells by showing the shift of cells from Lower Quadrant (FL1) to Upper Quadrant (FL2). This clearly suggests the suppression of the mitochondrial membrane damage or depolarization and confirms the protective nature of our formulation, FMT-SLNs-PS80.

### FMT-SLNs-PS80 protects against rotenone induced oxidative stress in SH-SY5Y cells

The oxidative markers such as ROS, SOD and CAT levels in SH-SY5Y cells treated with different treatment group were analysed using the standard curves plotted in different concentrations versus absorbance at 450 nm (Supplementary information Tables S10, S11, S12 and Figs. S16, S17, S18). Compared to Naïve FMT, the FMT-SLNs-PS80 showed greater protection against rotenone induced changes in ROS, SOD and CAT levels (Table 5).

**Table 5:** Quantitative estimation of oxidative markers (ROS, SOD and CAT levels).

Group	OD at 450 nm	ROS (ng/mL)	SOD (U/L)	CAT (U/L)
UT: Untreated cells	0.1108	3.17	126.432	1.656
S1: Rotenone (10 μΜ)	0.1417	4.09	106.934	0.446
S2: Rotenone + Naïve FMT (12.5 μg/mL)	0.1315	3.78	111.171	1.072
S3: Rotenone + FMT-SLNs-PS80 (69.5 μg/mL)	0.0969	2.75	120.352	1.685

### FMT-SLNs-PS80 decreases the SNCA expression in rotenone induced neurodegeneration in SH-SY5Y cell line

**Figure 8:**
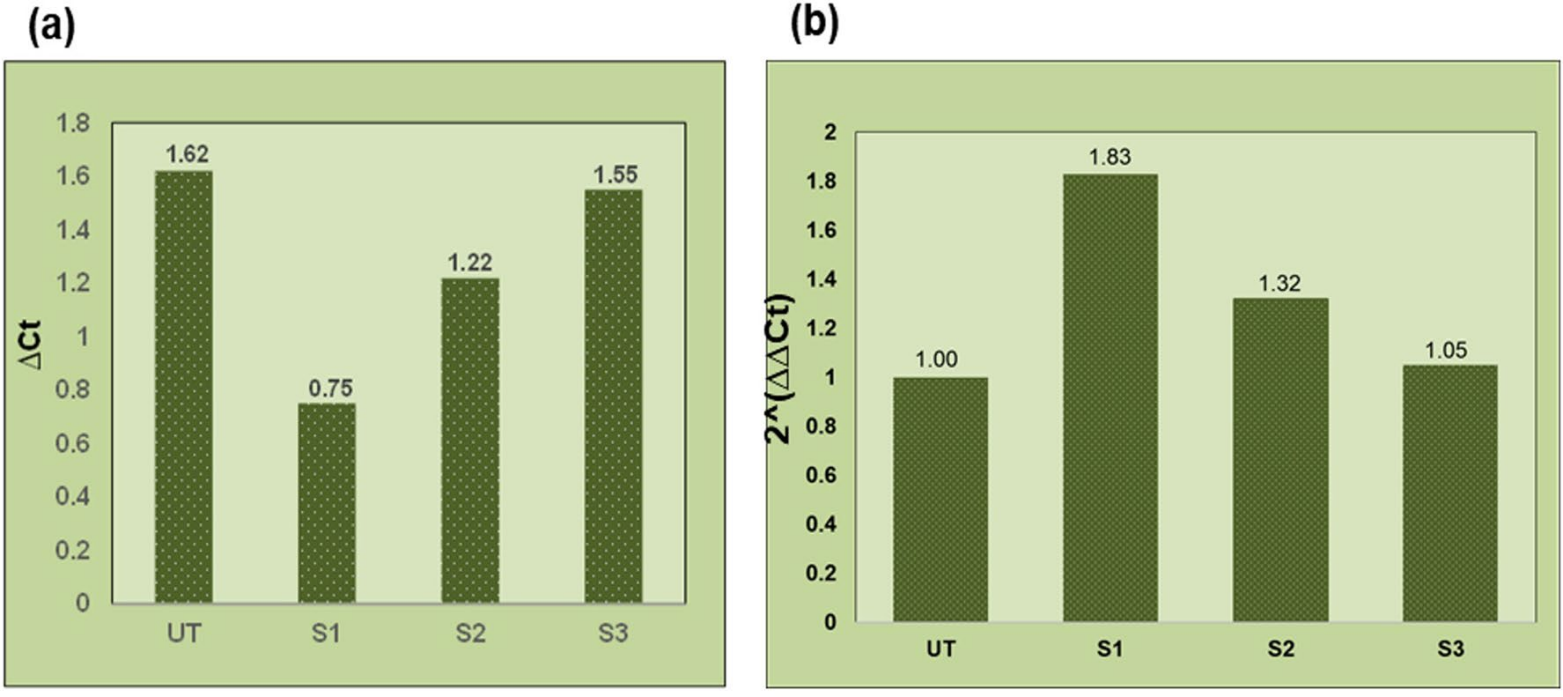
In vitro SNCA gene expression study using RT-PCR: (**a**) ∆Ct versus treatment groups, (**b**) 2^−(∆∆Ct)^ versus treatment groups. UT: Untreated cell, S1: Rotenone (10 μM), S2: Rotenone + Naïve FMT(12.5 μg), S3: Rotenone + FMT-SLNs-PS80 (69.25 μg).

The amount of target nucleic acid in the sample is inversely proportional to the Ct level, i.e. the lower Ct represents the target nuclei in the sample. Sampling in 96 well plate, melting curves and amplification cycle used in SNCA gene expression study is given in Supplementary information Fig. S19. The untreated group shows a Ct value of 1.62 which represents normal level of SNCA expression. However, exposure to rotenone in the S1 group showed a significant decrease in the Ct value (0.75) indicating a significant increase in SNCA gene expression. Pre-treatment with Naïve FMT (12.5 μg/mL) and FMT-SLNs-PS80 (69.25 μg/mL) significantly prevented rotenone induced changes in the Ct values (Fig. 8, Supplementary information Fig. S19, and Table S13).

### FMT-SLNs-PS80 protects rotenone induced neurodegeneration in PD models in vivo

The acute oral toxicity study of FMT-SLNs-PS80 was first performed in mice and all the six mice treated with FMT-SLNs-PS80 (2000 mg/kg p.o.) displayed no aberrant clinical indications and no mortality. All the animals gained weight and the gross necropsy analysis carried out at the end of the study revealed no abnormal findings. Thus, the acute oral LD_50_ of FMT-SLNs-PS80 was considered to be greater than 2000 mg/kg body weight.

**Figure 9:**
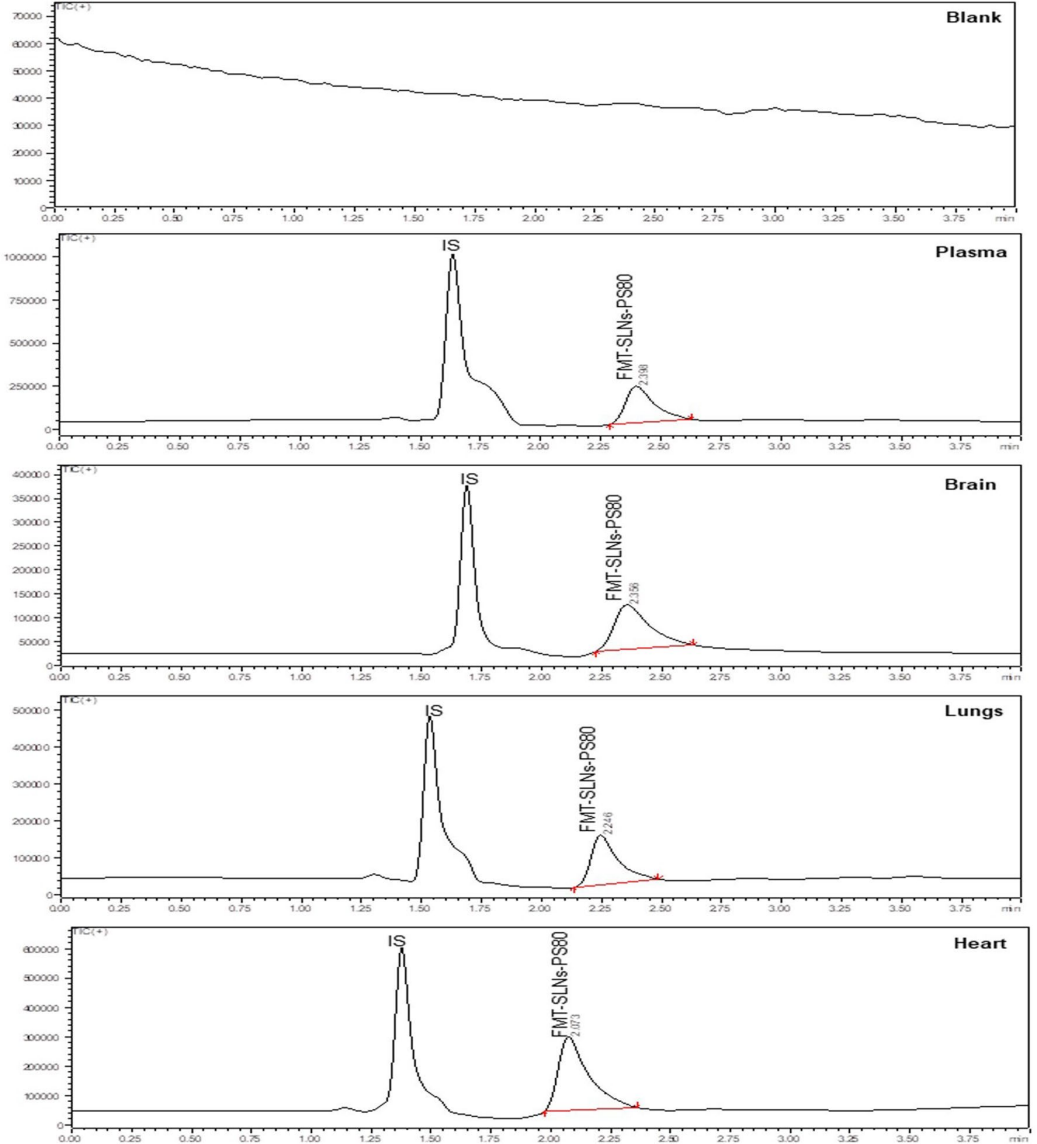
LC–MS chromatogram of FMT-SLNs-PS80 and IS (IBT) in various biological matrices.

**Figure 10:**
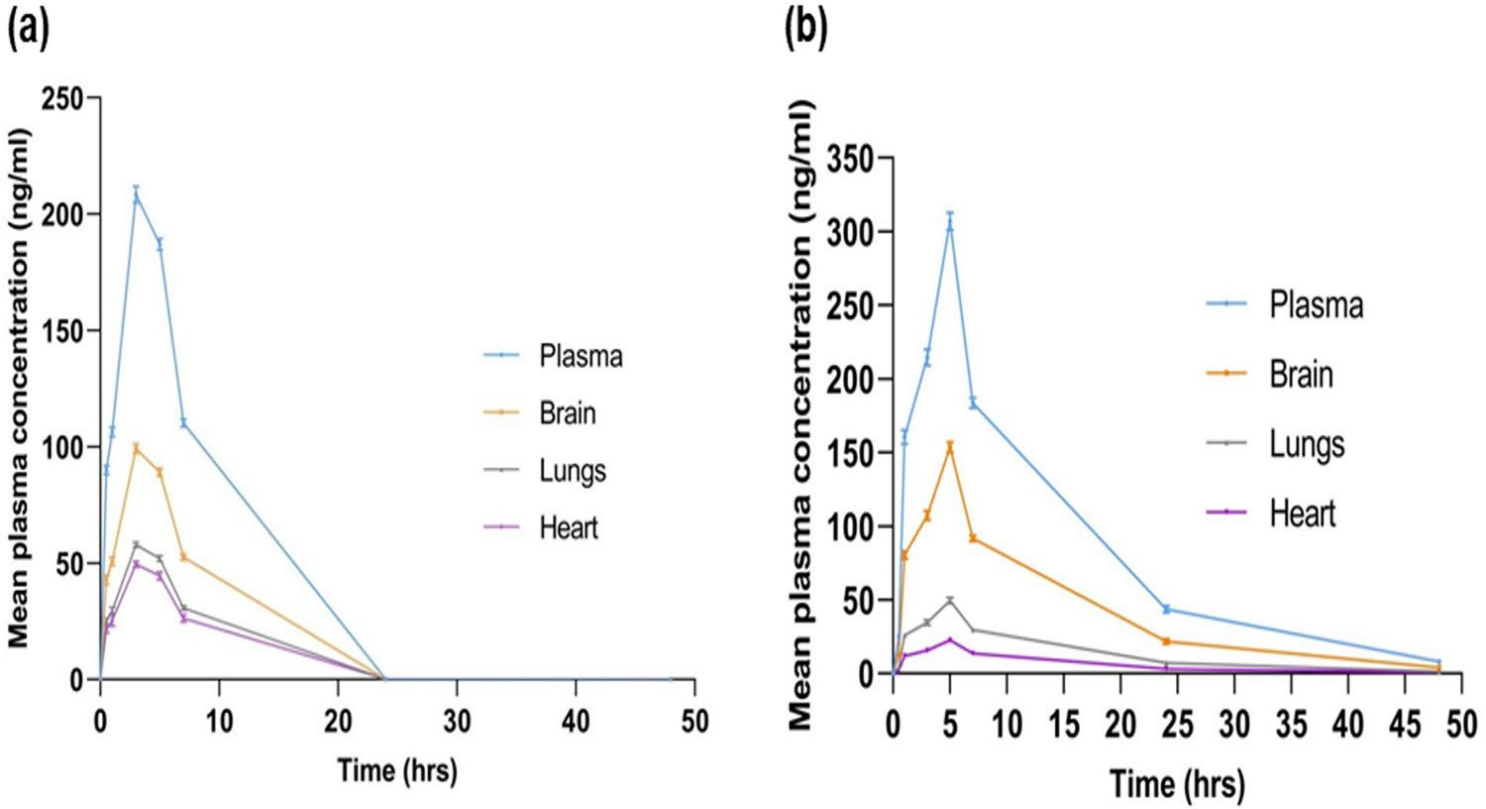
Pharmacokinetic profile: (**a**) naïve-FMT, (**b**) FMT-SLNs-PS80 in biological matrices.

The pharmacokinetics and tissue distribution profile following oral administration of FMT-SLNs-PS80 in rats reveal that in plasma, when compared to naïve FMT (AUC_(0–∞)_1771.2 ± 41.01 ng/h/mL), the FMT-SLNs-PS80 show 2.3 fold increase in plasma concentration (AUC_(0–∞)_ 4099.92 ± 54.13 ng/h/mL) and 2.4 fold increase in brain concentration (Naïve FMT AUC_(0–∞)_ 843.44 ± 31.91 ng/h/mL and FMT-SLNs-PS80 AUC_(0–∞)_ 2049.96 ± 22.72 ng/h/mL). Whereas, in peripheral tissues like lungs and heart there was 2.5 and 2.1 fold decrease in concentration (Naïve FMT lungs AUC_(0–∞)_ 492.01 ± 37.61 ng/h/mL, Naïve FMT heart AUC_(0–∞)_ 420.72 ± 19.51 ng/h/mL, FMT-SLNs-PS80 in lungs AUC_(0–∞)_ 200.28 ± 25.20 ng/h/mL, and FMT-SLNs-PS80 in heart AUC_(0–∞)_ 200.58 ± 28.20 ng/h/mL) (Figs. 9, 10). Thus, revealing the positive role of FMT-SLNs-PS80 for the brain specific delivery and therefore may redeem the peripheral side effect.

**Figure 11:**
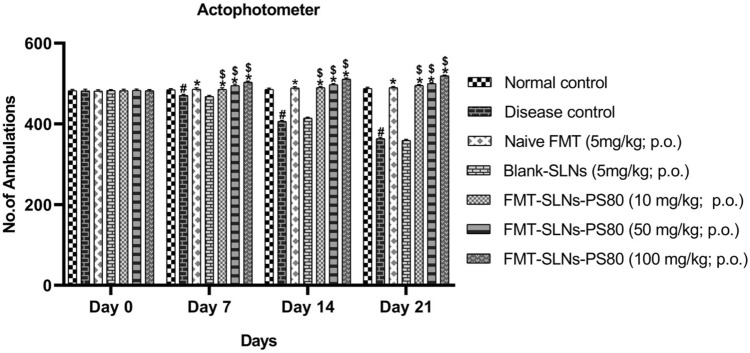
Locomotor activity in PD animal model. ^#^p < 0.05 versus normal control; *p < 0.05 versus disease control; ^$^p < 0.05 versus Naïve FMT; ^$^p < 0.05 versus Naïve FMT. The values are mean ± SD value (n = 6).

**Figure 12:**
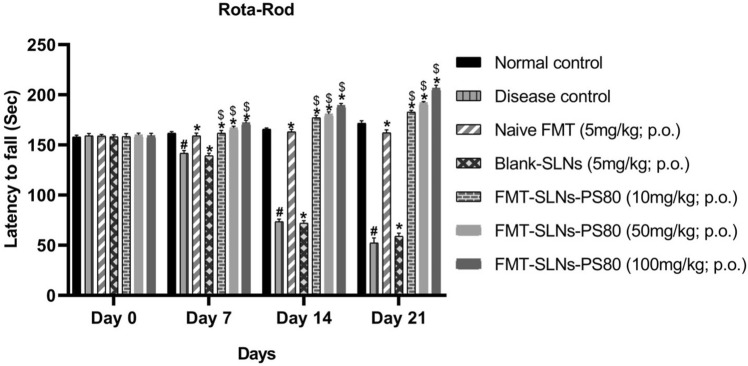
Muscle grip strength test in PD animal model. ^#^p < 0.05 versus normal control; *p < 0.05 versus disease control; ^$^p < 0.05 versus Naïve FMT; ^$^p < 0.05 versus Naïve FMT. The values are mean ± SD value (n = 6).

**Figure 13:**
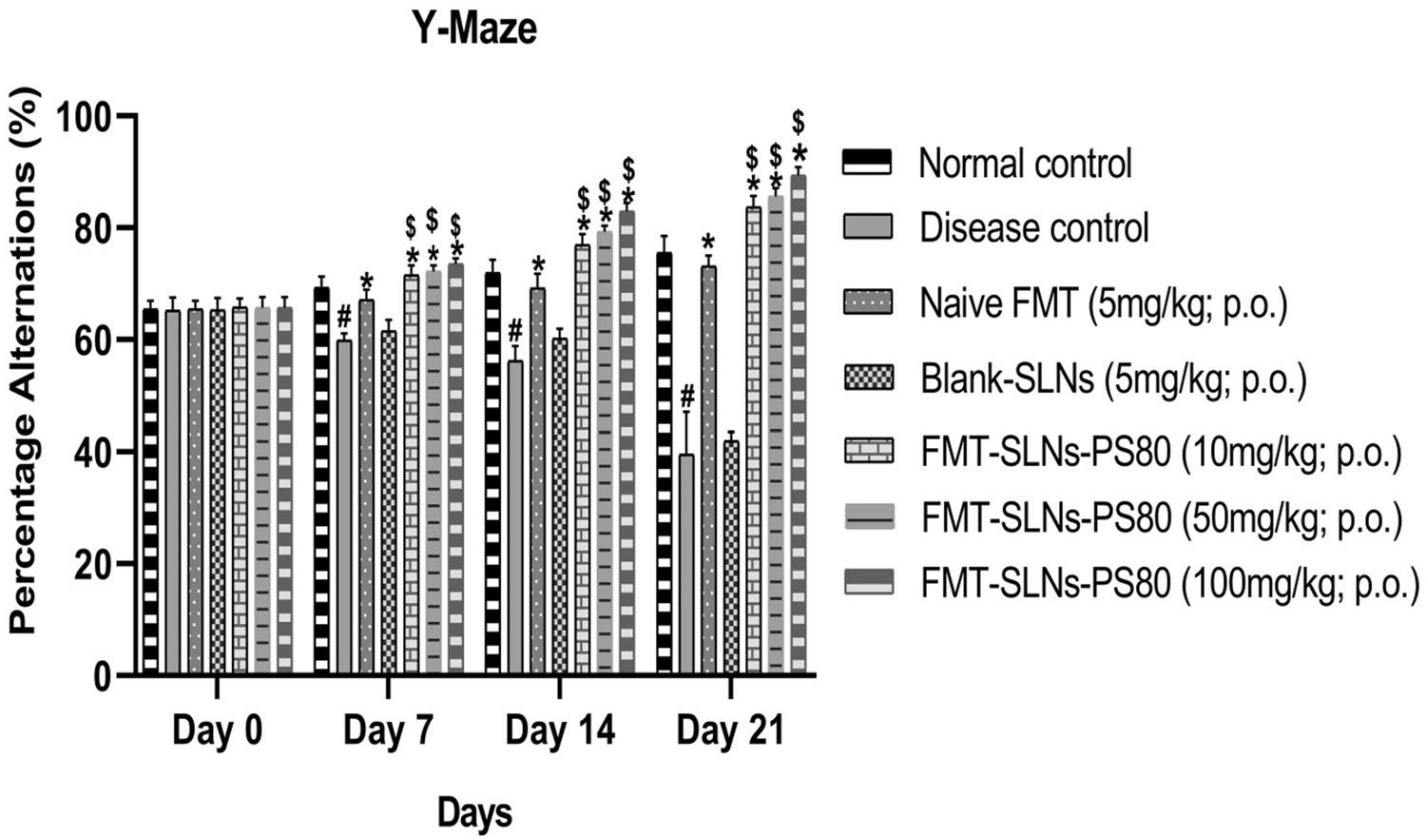
Memory function test in PD animal model. ^#^p < 0.05 versus normal control; *p < 0.05 versus disease control; ^$^p < 0.05 versus Naïve FMT; ^$^p < 0.05 versus Naïve FMT. The values are mean ± SD value (n = 6).

The prepared FMT-SLNs-PS80 was also evaluated for their anti-Parkinson’s efficacy in rotenone models in mice. Pre-treatment with FMT-SLNs-PS80 at 10, 50, 100 mg/kg, p.o. showed a significant dose reduction/prevention of rotenone induced changes in locomotor activity, muscle strength, and memory function (Figs. 11, 12, 13 respectively). Animals treated with naïve FMT (5 mg/kg, p.o.) show significant protection against rotenone induced decrease in behavioural study (p < 0.05). Animals treated with blank SLNs did not show any significant activity of rotenone induced changes.

The biochemical assessment showed that animals treated with naïve FMT (5 mg/kg p.o.) showed significant increase of levels SOD, CAT and GSH against rotenone induced decrease in SOD, CAT and GSH levels (p < 0.05). However, treatment with FMT-SLNs-PS80 at dose 10, 50 and 100 mg/kg, p.o showed significant (p < 0.05) dose dependent increase of rotenone induced decrease in the levels of SOD, CAT and GSH. Blank SLNs (5 mg/kg; p.o) treated animals did not show any activity in the rotenone induced changes in SOD, CAT and GSH levels (Table 6).

**Table 6:** Effect of naïve FMT, blank SLNs and FMT-SLNs-PS80 on SOD, CAT and GSH level in rotenone induced PD animal model.

Groups	SOD (units/mg protein)	CAT (units/mL)	GSH (μM/mg proteins)
Normal control	8.94 ± 1.23	2.97 ± 0.07	3.12 ± 0.18
Disease control	6.28 ± 0.54	0.62 ± 0.02	0.64 ± 0.09
Naïve FMT (5 mg/kg; p.o.)	7.54 ± 1.24	2.11 ± 0.12	2.15 ± 0.14
Blank SLNs (5 mg/kg; p.o.)	6.33 ± 1.07	0.83 ± 0.16	0.81 ± 0.18
FMT-SLNs-PS80 (10 mg/kg; p.o.)	7.83 ± 1.09	2.84 ± 0.09	2.58 ± 0.11
FMT-SLNs-PS80 (50 mg/kg; p.o.)	9.32 ± 1.13	4.12 ± 0.12	4.34 ± 0.13
FMT-SLNs-PS80 (100 mg/kg; p.o.)	12.21 ± 1.04	5.27 ± 0.16	5.66 ± 0.19

**Figure 14:**
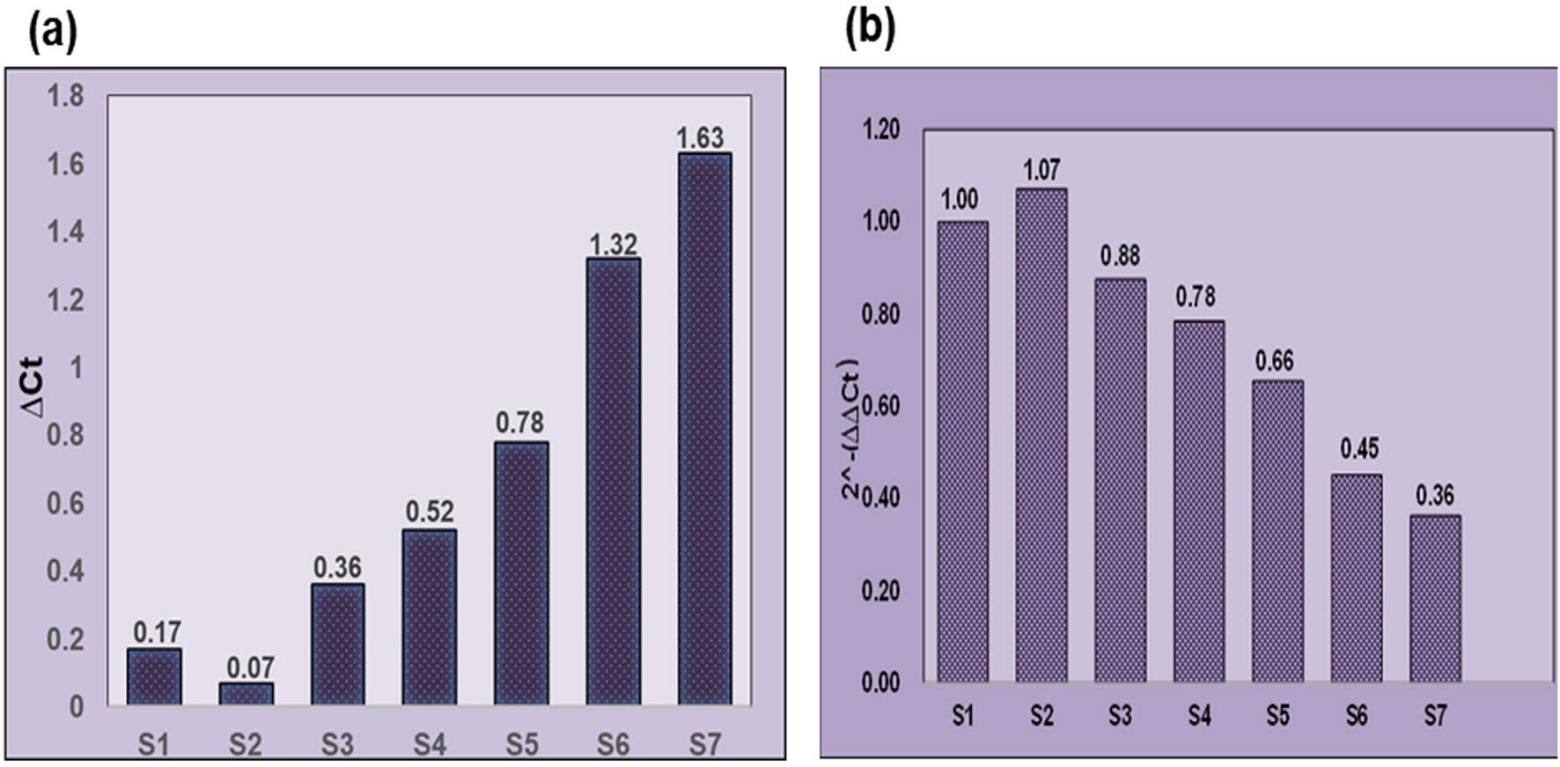
In vivo SNCA gene expression study using RT-PCR: (**a**) ∆Ct versus treatment groups, (**b**) 2^−(∆∆Ct)^ versus treatment group. S1: Normal control, S2: Disease control, S3: Blank SLNs (5 mg/kg; p.o.), S4: Naïve FMT (5 mg/kg; p.o.), S5: FMT-SLNs-PS80 (10 mg/kg; p.o.), S6: FMT-SLNs-PS80 (50 mg/kg; p.o.), S7: FMT-SLNs-PS80 (100 mg/kg; p.o.).

Further, the RT-PCR analysis and quantitative results are presented in Supplementary information Fig. S20. Sampling in 96 well plate, melting curves and amplification cycle used in SNCA gene expression study is given in Supplementary information Fig. S20. The normal control group, show a Ct value of 0.17 which represents normal level of SNCA expression. However, exposure to rotenone (2.5 mg/kg; p.o.) in disease control group (S2) showed a significant decrease in the Ct value (0.07) indicating a significant increase in SNCA gene expression. Pre-treatment with Naïve FMT (5 mg/kg; p.o.) and FMT-SLNs-PS80 (10, 50 and 100 mg/kg; p.o) significantly decreased the number of SNCA copies in rotenone induced changes in the Ct values in a dose-dependent manner (Fig. 14 and Table S17). Thus, depicting the capability of FMT-SLNs-PS80 in suppressing the SNCA expression was suppressed on treatment.

**Figure 15:**
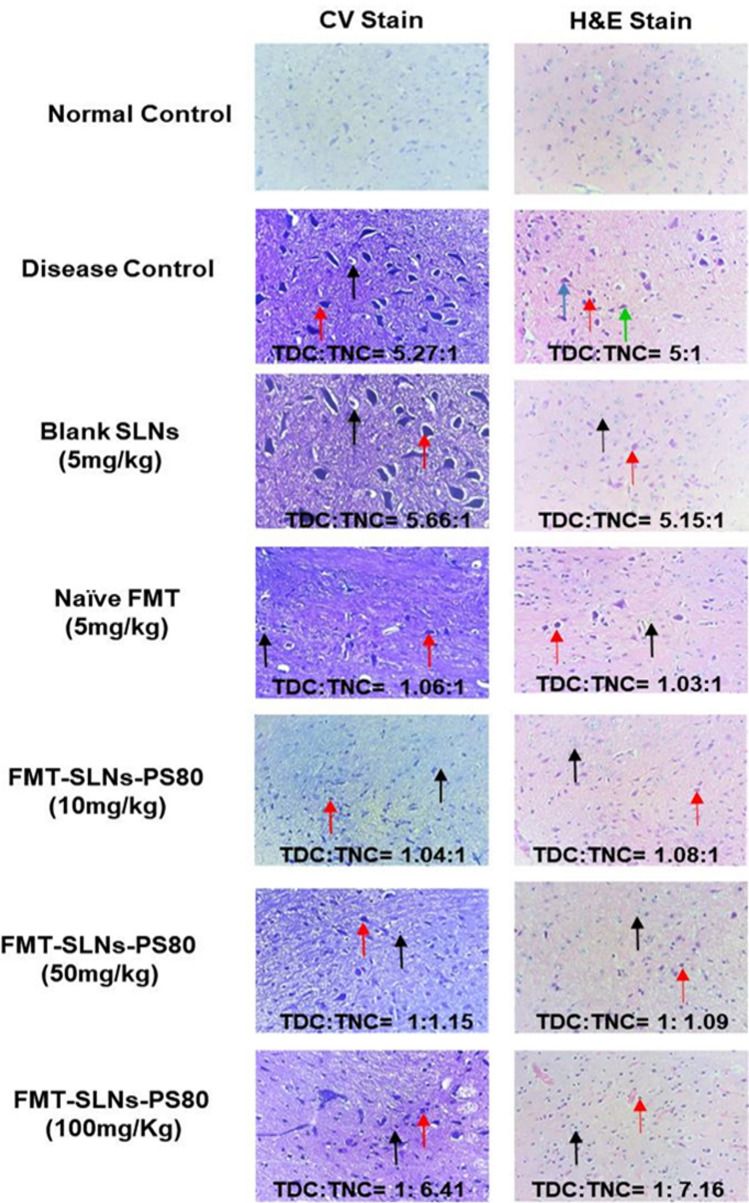
CV and H&E stain of substantia nigra of different treatment groups. *TDC* total number of degenerative neuronal cells, *TNC* total number of normal neuronal cells,  (red arrow): degenerative neuronal cells with darkly stained nucleus, ↑ (black arrow): nuclei of viable neurons,  (blue arrow): basophilic cytoplasm and neurofibrillary tangles,  (green arrow): focal reactive microgliosis.

In order to know the brain specific delivery of the formulation, the histological analysis of mice substantia nigra was performed. FMT-SLNs-PS80 treated group exhibited a dose dependent neuroprotection with increased number of normal neuronal cells and decreased number of degenerative neuronal cells in rotenone induced neurodegeneration in mice model (Fig. 15). Thus, reveals that FMT-SLNs-PS80 has comparable efficacy and site-specific delivery therefore may emerge as a potential neuroprotective agent and possibly halt neurodegeneration.

## Discussion

Research of SNCA in PD is of great interest as it is a major component in Lewy bodies, the protein clumps which is the pathological hallmark of PD^10^. Drug repurposing is an effective alternative drug development approach in therapeutic areas particularly to target psychiatric and neurological disorders. It is cost-effective and has helped to bridge the gap between rising therapeutic requirements and restricted drug discovery output^11^. Brain β2-receptor activation through β2-receptor agonists possibly regulate SNCA gene expression and thus could be repurposed as new anti-Parkinson drug^8^. SLNs based delivery system has been successfully employed for targeted delivery of drug as it permits high drug loading, high drug entrapment efficiency, good tolerance, site specific delivery, allows surface modification, thus improves bioavailability of various drugs^12^. In our study, for repurposing β2AR agonist, FMT as anti-Parkinson agent, an effort has been made to prepare and evaluate FMT as SLNs surface modified with PS-80 (FMT-SLNs-PS80) in order to achieve brain specific delivery and avoid the peripheral side effects for the management of PD.

For quantification of FMT in the formulation, method development was done using LC–MS which was found to be accurate, precise, and rapid. The developed method was validated for linearity, accuracy (recovery), precision (inter-day and intraday) studies as per USFDA guidelines, and was found to be within the acceptable limits, with a correlation coefficient, R^2^ of 0.9903 for linearity ranging between 20–250 ng/mL. The brain specific delivery of nano formulations is influenced by several characteristics like PS, ZP, shape and surface modification^13–15^. The prepared formulation, FMT-SLNs-PS80 yield PS of 151.28 ± 0.33 nm and ZP of 22.11 ± 3.18 mV, which falls in the desired range for brain specific delivery. PDI, the size-based measurement of heterogeneity in formulation was found to be 0.143 ± 0.11 in our optimized formulation, indicating the near monodispersity of the formulation. Furthermore, not only the size and overall surface area of NPs, but also the shape, has an impact on NPs receptor engagement and protein adsorption. Bartczak et al. reported that cellular uptake of particles of various shapes (spherical, rod-shaped, hollow, and silica-gold core–shell particles) differed and the spherical particles showed the highest uptake and hollow particles the lowest^16^. The surface morphology of FMT-SLNs-PS80 performed in SEM and TEM showed revealed the near spherical nature of the particles.

Surface chemistry or functionality aids for NPs BBB penetration. The functionalities can be classified into numerous categories such as surfactants and targeting ligands^17^. Surfactants facilitate the adsorption of blood proteins by engaging with receptors and/or transporters in the brain endothelium, allowing nanoparticles to pass over the BBB. Surfactants like PS80 and poloxamer 188, have been found to help nanoparticles deliver agents over the BBB. Surfactants on the surface of nanoparticles adsorb Apo A-I and/or Apo E from the blood, causing receptor-mediated endocytosis followed by transcytosis across the BBB^18,19^.

The in vitro drug release studies of naïve FMT and optimized FMT-SLNs-PS80 carried out using dialysis bag method showed very fast release of naïve FMT (96.54 ± 1.99% in 8 h). This could be owing to a lack of a nano-particulate system to control drug release. However, FMT-SLNs-PS80 were shown to release FMT over a time of 48 h with biphasic release and an initial burst release up to 6 h, followed by a controlled release up to 48 h (90.42 ± 3.07). The drug encapsulation patterns as well as the surface features of FMT-SLNs-PS80 may have an impact on the release behaviour. FMT-SLNs-PS80 is assumed to have core–shell structure with FMT absorbed in the shell which leads to an initial drug release. Thus, FMT in the shell release more quicker than in the core which can only be released slowly through dissolution and diffusion from the lipid matrix^20,21^. The drug release kinetics plots reveals that FMT-SLNs-PS80 follows first order release kinetics and obeys non-Fickian mode of drug release^22^.

The intestinal permeability plays a vital role in absorption of SLNs which depends on the content, size, charge and solubility. Ex vivo permeation study performed in everted chick ileum showed that FMT-SLNs-PS80 has a higher permeability when compared to naïve FMT solution. The P_app_ of naïve FMT calculated for the transport of naïve FMT solution was compared with FMT-SLNs-PS80. The result exhibited that FMT-SLNs-PS80 has higher P_app_ (5.70 × 10^−5^ cm/min) as compared to naïve FMT solution (1.37 × 10^−5^ cm/min). The increased permeability of the FMT-SLNs-PS80 may be due to SA which was used as lipid in the formulation. The increased permeability may be due to PS80 which was used as surfactant and surface modifying agent in the formulation. PS80 is reported to inhibit the intestinal P-gp thus increasing the permeability and may aid in the absorption of the FMT as they are capable of modifying membrane permeability and hence improving the absorption across the gut^9,23^. The increased permeability of the FMT-SLNs-PS80 may also be due to their submicron size, which may enhance contact surface area and prolong drug residence time thus substantially responsible for the improvement in intestinal permeability^24–26^.

The mitochondrial membrane potency effect of naïve FMT and FMT-SLNs-PS80 was performed on SH-SY5Y cell lines pre-induced with 10 μM of Rotenone. FMT-SLNs-PS80 showed more significant reduction of mitochondrial membrane as compared to naïve FMT. FMT-SLNs-PS80 also attenuated rotenone induced changes in oxidative stress parameters such as ROS, SOD and CAT levels in rotenone induced SH-SY5Y cells. FMT-SLNs-PS80 decreased the number of SNCA copies in rotenone induced increased SNCA expressions in SH-SY5Y cells.

**Figure 16:**
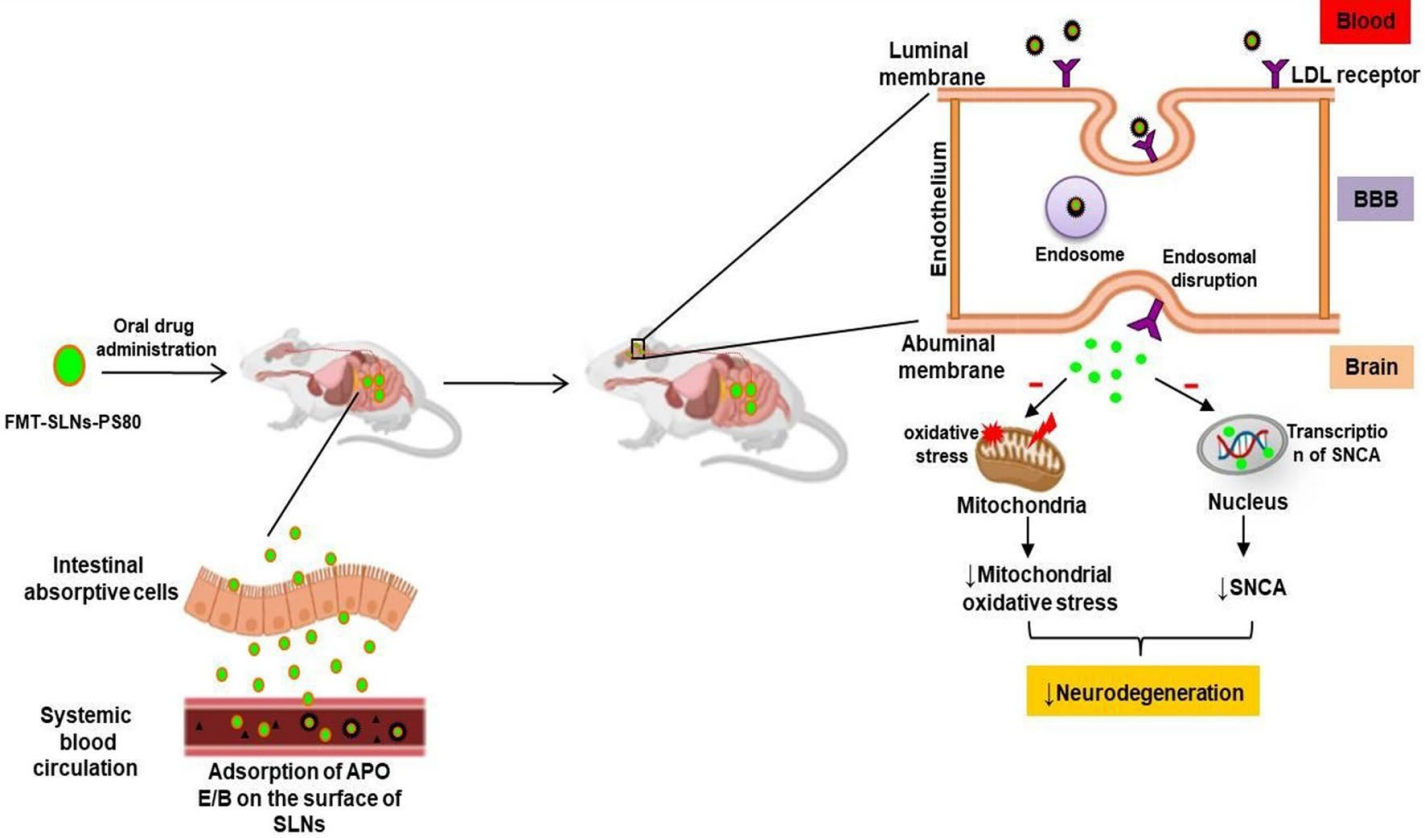
Schematic representation of the fate of FMT-SLNs-PS80 through oral delivery.

The pharmacokinetics and tissue distribution studies following oral administration of FMT-SLNs-PS80 in rats showed low deposition of FMT in peripheral tissues (lungs and heart) and higher accumulation in the brain as compared to naïve FMT. Hence, depicting the brain specific delivery of FMT-SLNs-PS80 and redeeming of the peripheral side effect, and as a result, the successful repurposing of the existing β2AR agonist, FMT for the treatment of PD. The increase in deposition of FMT from FMT-SLNs-PS80 may be due to PS80 which was employed as a surface modifying agent during the formulation. Several studies have reported that PS80 facilitates the adsorption of apolipoprotein E or B from the blood on the NPs surface allowing the particles to imitate the endogenous transport of LDL and activates the LDL receptor-related proteins (LRP-1 and LRP-2) which are expressed on the BBB^27,28^. Thus apolipoproteins absorbed NPs are endocytosed by capillary endothelial cells and delivered into the brain via the BBB by receptor-mediated endocytosis^29,30^. Further, PS80 are reported to inhibit the outflow of the P-gp pump. The P-gp transporter is particularly concentrated at the BBB which prevent the exogenous drug penetration into the brain^29,31^. These pathways may operate in parallel or in co-operation, allowing for drug transport to the brain^27^. The schematic representation of the fate of FMT-SLNs-PS80 through oral delivery is depicted in Fig. 16.

The prepared FMT-SLNs-PS80 evaluated for their anti-Parkinson’s efficacy in rotenone models in mice showed a significant dose dependent reduction of rotenone induced changes in locomotor activity, muscle strength, and memory, and also levels of CAT, SOD and GSH. FMT-SLNs-PS80 also decreased the number of SNCA copies in rotenone induced increased SNCA expressions in mice. The histological sections of substantia nigra of FMT-SLNs-PS80 treated group revealed increased number of normal neuronal cells and decreased number of degenerative neuronal cells in ROT induced neurodegeneration in mice model. Thus, depicts the effectiveness and site-specific delivery of FMT-SLNs-PS80, therefore may emerge as a potential neuroprotective agent and possibly halt neurodegeneration.

**Figure 17:**
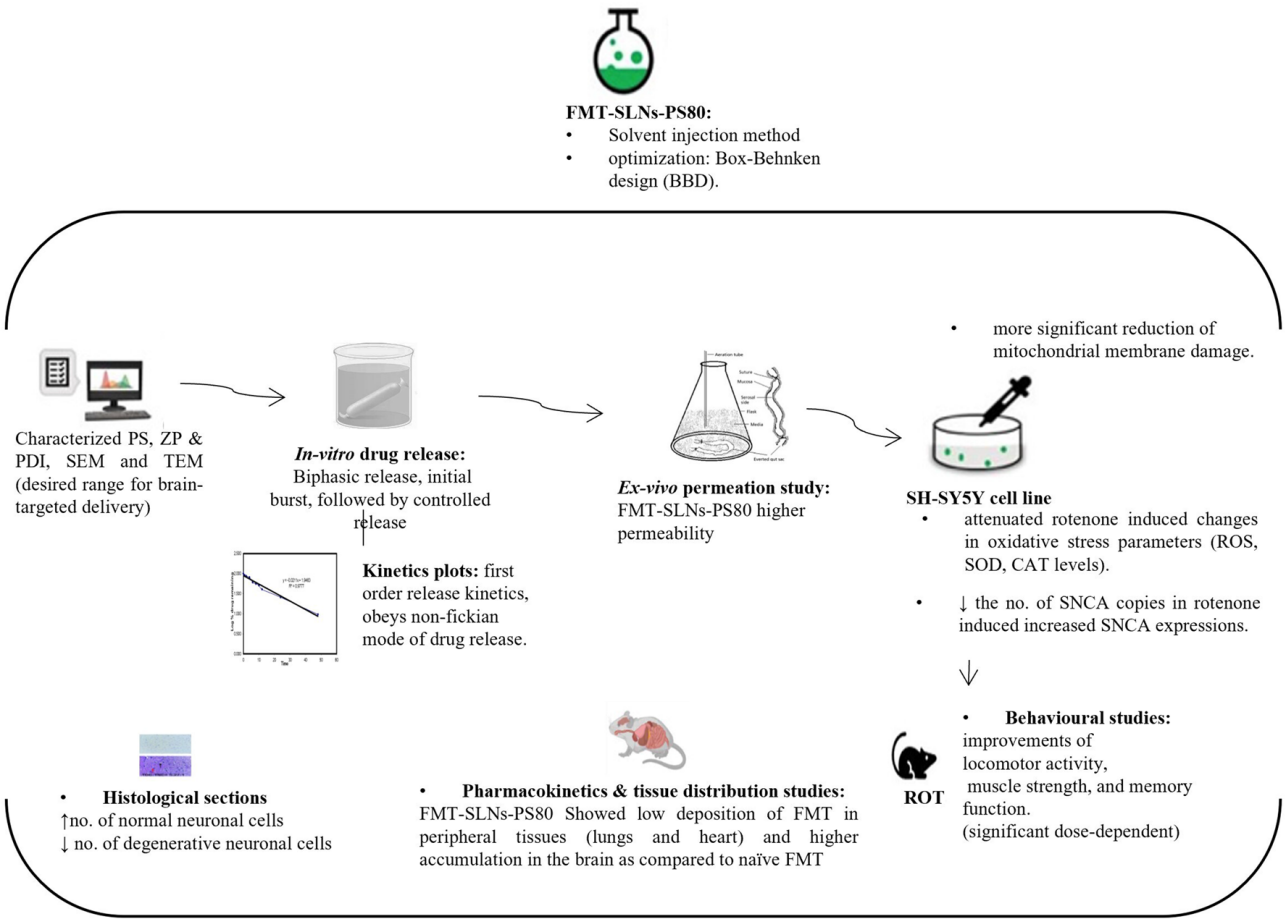
Neuroprotection of FMT-SLNs-PS80 in in vitro and in vivo rotenone models of PD.

Altogether, the FMT-SLNs-PS80 formulation exhibited significant neuroprotective benefits in both in vitro and in vivo models of PD and may be a potential candidate for treating neurodegeneration (Fig. 17). The developed formulation may help to minimize or eliminate the peripheral side effects and may also significantly slow down the progression of PD. Further development of the formulation may help millions of PD patients to better manage the disease with improved patience compliance.

## Methods

### Pre-formulation studies

The compatibility of FMT and SA was checked for the thermal stress stability using DSC. DSC thermograms were analyzed of pure drug (FMT), SA and their physical mixture (FMT + SA, 1:1). Alumina was used as a reference standard. 1 mg of sample was packed in aluminium cells before being placed in a Mettler TA 4000 thermal analyser. The thermal study was carried out in a nitrogen environment at a heating rate of 100 °C per minute across a temperature range of 30–300 °C.

The molecular and chemical compatibility was checked by FT-IR^32^. The compatibility between the FMT, SA and their physical mixture (FMT + SA, 1:1) was checked FT-IR. KBr pelletization was done and scanned using an FT-IR spectrophotometer at IR range 4000–400 cm^−1^.

### Design and optimization of FMT-SLNs-PS80 using Box–Behnken design

The solvent injection approach was used to make a trial batch of FMT-SLNs-PS80, which was then sonicated. As a lipid, SA was used, methanol as solvent and PS80 as surfactant and surface modifying agent. Briefly, FMT (5 mg) and SA (45 mg) were dissolved in 3 mL of methanol (lipid phase). The lipid phase was quickly injected to aqueous solution containing 4% PS80 and was stirred on a magnetic stirrer for 45 min. The resulting emulsion was probe sonicated at 80 amplitude, 0.6 cycles for 6 min. The formed nano-dispersion was stirred to evaporate the solvent and allowed to cool to room temperature. Similarly, blank SLN formulation was prepared by excluding drug.

Optimisation of FMT-SLNs-PS80 formulation was done using three factors and three levels Box–Behnken design (BBD) with three central points and 15 experimental runs using Minitab^®^18.1 statistical tool. The surfactant (PS80) concentration (%w/v) (A), sonication amplitude (%) (B), and the sonication time (min) (C) were employed as independent variables, at three different concentrations, − 1, 0, and + 1 which correspond to low, medium and high respectively. The dependent variables or responses were PS, and ZP (Table 7). A quadratic mathematical equation was used to validate the model. Further, 15 experimental batches were prepared and characterised based on the DoE software's recommendations. The data was then fed into the software and was used to optimise the formulation based on desirability criteria^33^.

**Table 7:** Variables and responses used for Box–Behnken design.

Factor	Levels		
Independent variables	Low (− 1)	Medium (0)	High (+ 1)
A: Surfactant concentration (%)	2	4	6
B: Sonication amplitude (% w/v)	60	80	100
C: Sonication time (min)	3	6	9
Dependent variables			
Particle size	Minimize		
Zeta potential	Maximize		

### Characterization of FMT-SLNs-PS80

The PS, ZP, and PDI of FMT-SLNs-PS80 were measured using Nano-Brook (90PLUS Zeta). The PS and PDI were calculated using a photon correlation spectroscopic approach to analyse variations in dynamic light scattering (DLS). The surface charge (ZP) of the SLNs formulation was measured by an electrophoretic light scattering method. After dilution of all samples with distilled water, disposable polystyrene cells and disposable plain folded capillary zeta cells were employed for PS, PDI, and ZP measurements, respectively. All measurements were repeated three times and the findings were expressed as the particle mean size ± SD^34^.

The surface morphology of the FMT-SLNs-PS80 was characterized by scanning electron microscopy (SEM) and transmission electron microscopy (TEM) (S2.1). DSC and FT-IR analysis of FMT-SLNs-PS80 was performed following the same procedure as mentioned earlier (section Pre-formulation studies). The results were compared with the naïve drug to confirm the drug being entrapped.

The drug content, drug loading (DL) and entrapment efficiency (EE) in formulation were determined. To determine the drug content, FMT-SLNs-PS80 (1 mg) was dissolved in 10 of methanol and vortexed for 5 min at 4500 rpm for 5 min followed by appropriate dilution and analysed by the developed LC–MS method mentioned in S1.1^35^.

The DL and EE in formulation was determined by centrifuging FMT-SLNs-PS80 dispersion at 15,000 rpm for 20 min. The concentration of free FMT in the supernatant was measured by the developed LC–MS method (refer S1.1). The percentage DL and EE were calculated by the following formulae^35^.

$${\text{DL}}\;(\% ) \, = \frac{{{\text{Total}}\;{\text{FMT}}\;({\text{mg}})\;{\text{added}} - {\text{Total}}\;{\text{FMT}}\;{\text{in}}\;{\text{supernatant}}}}{{{\text{Amount}}\;{\text{of}}\;{\text{SA}}\;{\text{added}}\;({\text{mg}})}} \times 100$$

$${\text{EE}}\;(\% ) \, = \frac{{{\text{Total}}\;{\text{FMT}}\;({\text{mg}})\;{\text{added }} - {\text{Total}}\;{\text{FMT}}\;{\text{in}}\;{\text{supernatant}}}}{{{\text{Amount}}\;{\text{of}}\;{\text{FMT}}\;{\text{added}}\;({\text{mg}})}} \times 100$$

### In vitro drug release studies for optimized batch of FMT-SLNs-PS80

The dialysis bag method was used to investigate the in vitro release of FMT from the optimised batch of FMT-SLNs-PS80. The experiment was carried out on nanoparticle suspension within 24 h of preparation utilising a dialysis membrane of molecular weight cut off 12,000 Da. The FMT-SLNs-PS80 dispersion or naive FMT solution (equivalent to 5 mg of FMT) was dialyzed against 500 mL of dissolution medium (artificial cerebrospinal fluid, Acsf, pH 7.4) and agitated at 100 rpm and kept at 37 ± 0.5 °C. The loss by evaporation was minimised by covering the beaker with aluminium foil. Samples (500 µL) were taken at intervals of 0.5, 1, 2, 4, 6, 8, 10, 12, 24, and 48 h and evaluated for drug content using the developed LC–MS method. At each time point, the sink condition was maintained by adding fresh dissolution medium. All measurements were taken three times and the results were calculated using the total amount of FMT released^36,37^.

### aCSF composition

Solution-I: Sodium chloride (8.66 g), calcium chloride (0.206 g), potassium chloride (0.224 g), and magnesium chloride (0.163 g) in 500 mL distilled water.

Solution-II: Disodium hydrogen phosphate (0.214 g) and sodium dihydrogen phosphate (0.027 g) in 500 mL distilled water.

Solution-I and II were mixed in a ratio of 1:1^38^.

The obtained release profiles were fitted into various kinetics models such as zero order, first order, Higuchi, Korsmeyer–Peppas and Hixon–Crowell models. The correlation coefficient between cumulative percentage of drug release and time were calculated to find the suitable kinetics. The mechanism of drug release was evaluated by exponent n-value in the slope of the straight line from Krosmeyer–Peppas equation^39^.

### Ex vivo permeation studies for optimized batch of FMT-SLNs-PS80

Ex vivo permeation was studied by using everted chicken ileum model^40^. Briefly, the ileum was divided into 5–6 cm segments and washed with ice-cold Krebs solution [pH 6.5, composition—sodium chloride (7 g/L), potassium chloride (0.34 g/L), glucose (1.8 g/L), disodium hydrogen phosphate (0.251 g/L), sodium dihydrogen phosphate (0.207 g/L) and magnesium chloride (46.8 mg/L)]^40^.

The washed intestine was gently everted over a glass rod. A silk braided suture was used to secure one end of the everted intestine, which was subsequently filled with 600 mL Krebs solution at 37 °C using 1000 μl micropipette^41,42^. Another braided silk suture was used to tie a second knot around the filled intestinal section. Then, it was transferred to the incubation flask maintained at 37 °C containing Naïve FMT/FMT-SLNs-PS80 in 15–25 mL oxygenated media. To check the absorption of Naïve FMT/FMT-SLNs-PS80 at different time intervals (0, 60, 90 and 120 min), different labelled flask was used. The flasks were incubated under continuous shaking and gassing with 95% oxygen–5% carbon dioxide in orbital water bath shaker maintained at 37 °C throughout the incubation period. After incubation, everted sac was removed from the incubation bath and opened at one end. The contents of the sac (serosal solution) were drained and kept in a labelled test tube^10,11^. The FMT concentration present in serosal solutions was measured using the developed LC–MS method.

Apparent permeability coefficient (P_app_) was measured by using equation^41^,

$${\text{P}}_{{{\text{app}}}} = {\text{ F }} \times \, 1/{\text{A}}.{\text{C}}_{0} \;{\text{cm}}\;{\text{min}}^{ - 1} ,$$

where, F represents the permeation flux (ng min^−1^) which is calculated by plotting the cumulative amount of FMT permeated (ng) through the sac against time (min). A is the membrane’s surface area (cm^2^) and C_0_ represents the FMT initial concentration.

### Cell culture

SH-SY5Y human neuroblastoma cell line was grown in Dulbecco’s Modified Eagle’s Medium (DMEM) containing 10% Fetal Bovine Serum.

### In vitro neuroprotective activity

### Cytotoxicity studies

The MTT assay was used to test the cytotoxicity on SH-SY5Y (human neuroblastoma) cell line (Supplementary information S2.4).

#### Evaluation of mitochondrial membrane potential

The mitochondrial membrane potential (ΔΨM) was measured using JC-1 (5′,6,6′-tetrachloro-1,1′,3,3′-tetraethyl benzimidazol carbocyanine iodide), a lipophilic cationic dye which naturally exhibits green fluorescence (Supplementary information S2.5).

#### Estimation of oxidative markers

ROS, SOD and CAT were determined by using ELISA kit (Supplementary information S2.6, S2.7 and S2.8 respectively).

#### In vitro SNCA expression studies (RT-PCR)

After the treatment, total RNA was extracted from cells using Trizol based RNA Extraction (Supplementary information S2.9). CFX Opus 96Dx, Real-Time PCR System and QIAqurant 96 Splex were used to evaluate the relative SNCA mRNA levels. Refer S2.9 for cDNA synthesis, primer sequences (Supplementary information Table S18), PCR reaction mixtures and conditions (Supplementary information Table S19).

### In vivo studies

#### Animals

Adult male Swiss albino mice (25–30 g) and male Wistar rats (180 ± 20 g) were maintained in the central animal house of JSS College of Pharmacy, Ooty. Standard laboratory conditions were maintained (22 ± 2 °C, 12 h light and dark cycle) for the animals with normal pellet diet and free access to water. The experiments were carried out with prior approval from the Institutional Animal Ethics committee (IAEC), JSS College of Pharmacy, Ooty (Approval Number: JSSCP/IAEC/OT/PhD/28/2018-19). All the methods in the study were performed as per the CPCSEA guidelines for laboratory animal facilities. The reporting of the studies was done in accordance with ARRIVE guidelines.

#### Test item preparation

For in vivo studies, the FMT, FMT-SLNs-PS80 and blank SLNs were prepared as a suspension in 0.5% w/v carboxy methyl cellulose (CMC) at 1/10th concentration of the desired dose and administered at a dose volume of 10 mL/kg body weight.

#### Acute oral toxicity of FMT-SLNs-PS80

Acute oral toxicity of FMT-SLNs-PS80 was studied in Swiss albino mice following OECD 423 Guidelines. Six mice were used in a limit test at a dose of 2000 mg/kg, p.o. (3 per step). Following overnight fasting the mice were administered with the test compound and were examined for clinical signs and mortality on daily basis for 14 days. Weekly body weight of all the mice was noted. On day 15, all the mice were culled by deep isoflurane anaesthesia and gross necropsy was performed.

#### Pharmacokinetic and tissue distribution studies in rat

After overnight fasting, 30 male Wistar rats were randomly divided into three groups, each with ten animals—control, Naïve FMT and FMT-SLNs-PS80 groups, followed by oral administration of 0.5% CMC, Naïve FMT (25 mg/kg) and FMT-SLNs-PS80 (140 mg/kg) and respectively (Supplementary information Table S20). Approximately 1 mL of blood was collected from retro-orbital plexus at 0, 0.5, 1, 3, 5, 7, 24 and 48 h in a time staggered manner (Supplementary information Table S20). One animal was culled at each time point and the brain, heart, and lungs were collected. The tissues were washed with 0.9% sodium chloride solution, blotted on filter paper and weighed. The tissues were then homogenized with normal saline at 1:4 (weight/volume) and stored at − 20 °C until further analysis. The extractions of FMT from plasma and tissues samples were done following the method described in Supplementary information S.1.1. The concentration of FMT from the samples was measured using the developed LC–MS method (Supplementary information S1.1). Further, the pharmacokinetic data was analysed using PK solver software.

### Anti-Parkinson’s activity in mice

#### Grouping of animals

**Figure 18:**
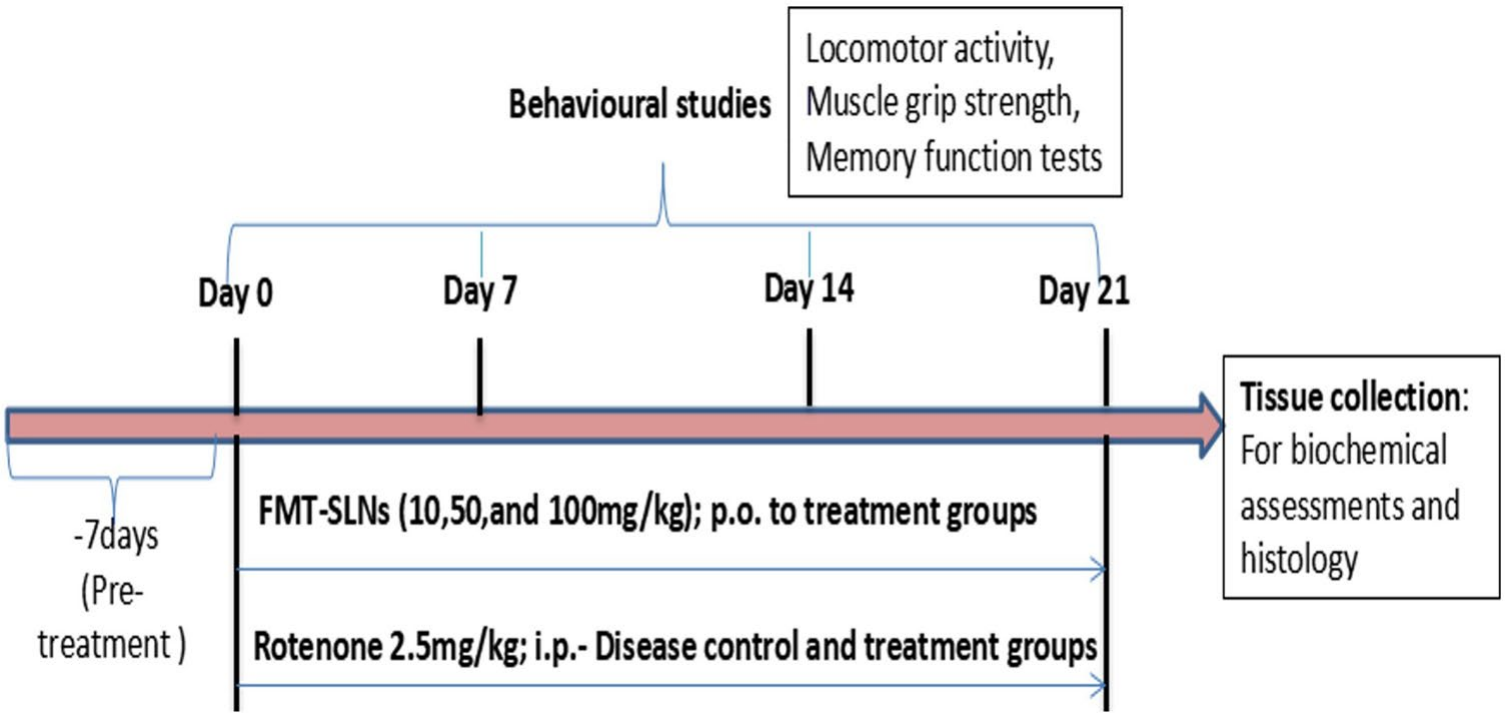
Design of experimental schedule.

Forty-two male mice were divided into 7 groups with 6 animals each (Table 8). Group-I and II served as normal and diseased control respectively and received vehicle (Carboxymethyl cellulose, CMC 0.5%) 10 mg/kg. Group III animals received naïve FMT at a dose of 5 mg/kg p.o. Group IV animals received blank SLNs at a dose of 5 mg/kg p.o. Group V, VI and VII animals received FMT-SLNs-PS80 at a dose of 10, 50 and 100 mg/kg respectively. All the animals received the assigned treatment for a period of 7 days before the exposure to rotenone (Fig. 18).

**Table 8:** Animal grouping.

Group	Treatment
I	Normal control (0.5% CMC 10 mL/kg; p.o)
II	Disease control (0.5% CMC 10 mL/kg; p.o)
III	Naive FMT (5 mg/kg; p.o.)
IV	Blank-SLNs (5 mg/kg; p.o.)
V	FMT-SLNs-PS80 (10 mg/kg; p.o)
VI	FMT-SLNs-PS80 (50 mg/kg; p.o)
VII	FMT-SLNs-PS80 (100 mg/kg; p.o)

#### Induction of PD

PD was induced in mice pre-treated for 7 days with assigned treatments. To induce PD, all groups except Group-I (normal control) received rotenone at a dose of 2.5 mg/kg i.p. one hour after their assigned treatments for a period of 21 days (Fig. 18). The first day of rotenone administration is considered as Day 0 of the study.

Behavioural studies such as locomotor activity, muscle grip strength and memory function test were performed on day 0, 7, 14, and 21 in all the groups (Fig. 18). At the end of the study (day 22), animals were anaesthetised with deep isoflurane anaesthesia, the abdominal was opened and the abdominal artery was punctured to drain the blood. The brain was collected for biochemical analysis and histopathological studies (Fig. 18).

#### Behavioural studies

The following behavioural parameters were assessed^43^.

#### Locomotor activity

A digital actophotometer was used to detect locomotor activity in mice. Mice were given for 1 min acclimatisation within the activity box. The number of photo beam interruptions was counted for 5 min to measure the locomotor activity.

#### Muscle grip strength

The muscular in-coordination was assessed using a rota-rod apparatus. Animals were trained for two consecutive days at a speed of 8 rpm on the 1st day and 10 rpm on the 2nd day and the rotation speed was increased to 15 rpm on the 3rd day. The time for each mouse spent on the rotating bar was recorded and the cut-off time was kept 180 s per trial. Automatic apparatus was used to record the time and the counting was stopped when the mice fell of the rotating shaft. To avoid error, the test was repeated three times and the average data were noted as retention time on the rotating bar.

#### Evaluation of memory function

The effect of FMT-SLNs-PS80 on rotenone induced memory deficits was assessed using the Y-maze^17^. Each mouse was placed at the end of one arm and was permitted to explore freely for 5 min in all the three arms. The number of arm visits and sequence (alternation) of arm visits were recorded. The percentage alternation is utilised as a spatial memory index. Consecutive entries into all the three arms (i.e. ABC, CAB or BCA but not BAB) were counted as alternation behaviours^44^.

### Biochemical studies^43^

#### Estimation of oxidative markers

The oxidative markers such as SOD and CAT were determined by the approach developed by Beyer and Fridovich and Aebi technique respectively (Supplementary information S2.10).

GSH level was determined by taking homogenised tissue in PBS (1 mL) in a test tube. 0.5 mL of phosphate buffer (0.2 M pH 8), 1.3 mL distilled water and 0.2 mL of 5,5′-dithio-bis (2-nitrobenzoic acid) (DTNB, 0.6 mM) were added. The components were thoroughly mixed and measured using spectrophotometer at 420 nm. The reduced GSH level was compared with the standard reduced glutathione graph.

### In vivo SNCA expression studies (RT-PCR)

#### Homogenization

Harvested brain tissues were homogenised using the homogeniser for 5 min with SS beads. Further, the homogenised samples were extracted for RNA, cDNA synthesis, for preparation of primer sequences, and performing PCR (as mentioned in Supplementary information S.2.9).

#### Histopathological analysis

Brain samples were collected and fixed in a 10% neutral formalin buffer for 24 h. After which, the brains were embedded into paraffin molds and sectioned into 8 μm thickness. Haematoxylin and eosin (H&E) and Cresyl Violet (CV) were used to stain the sections on glass slides. A high-powered field microscope was used to view the slides.

### Supplementary Information

Supplementary Information.

## Data Availability

Upon reasonable request can be obtained from the Corresponding author.
