# Supplementary material for: Polysorbate 80 surface modified SLNs of formoterol suppress SNCA gene and mitochondrial oxidative stress in mice model of Parkinson’s disease
Source: Sci Rep. 2023 Nov 15;13:19942. doi: 10.1038/s41598-023-46511-3 (PMC10651909; doi:10.1038/s41598-023-46511-3)
Supplement: Supplementary file 1 — Supplementary Information. [file 41598_2023_46511_MOESM1_ESM.docx]

Supporting Information

S1.1 LC-MS method development and validation

Chromatographic condition

The LC-MS method was designed for estimating FMT by electrospray ionization (ESI) technique. The LC-MS analysis of FMT was performed using Shimadzu 8020 Auto Sampler with LC-20-AD solvent delivery system pump, MS-detector, Lab solutions data processor, ESI interface, SIM operation mode. Zorbax C8 column (75mm x 4.6 mm) and selected mobile phase (acetonitrile and ammonium acetate 10Mm, pH 3.5, 80:20, v/v) was used for separation at the flow rate of 0.8 ml/min. The detection of FMT and internal standard (IS), Irbesartan (IBT) was achieved by using ESI technique. The working parameters such as block temperature of 250°C, and desolvation line temperature of 200°C, ambient probe temperature, detector voltage of 1.3kV, nebulizer gas flow of 1.5L/min, drying gas flow of 15 L/min, dwell time of 100 msec were maintained.

The mass scan spectra of FMT and Irbesardan (IS, internal standard), and their standard chromatograms are given in Figure S1 and Figure S2 respectively. The method shows linearity in 20-250 ng/ml range with FMT and IS retention time of 2.2 min and 1.5 min respectively (Figure S3). The optimized LC-MS conditions employed for the detection of FMT and IBT (IS) are given in Table S1.


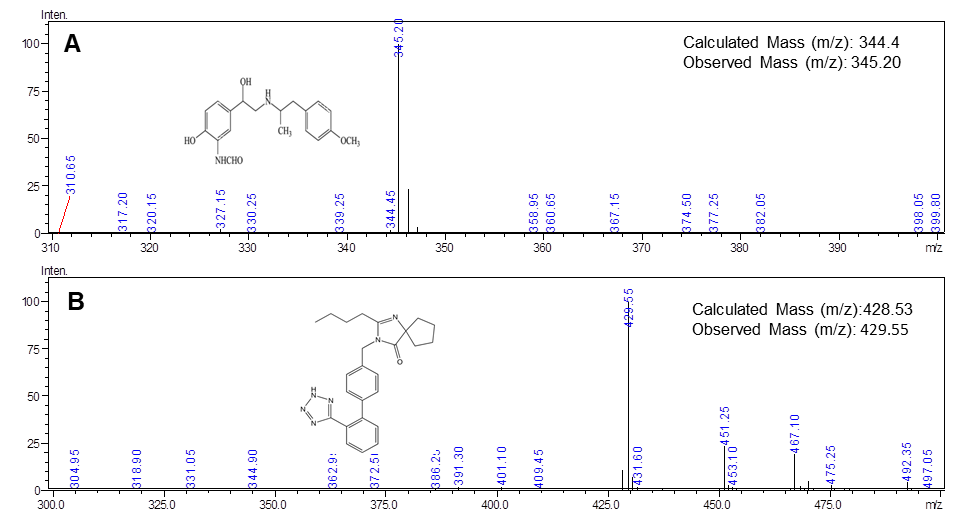


**Figure S1. Mass scan spectra of (A) FMT (B) Irbesardan (IS)**


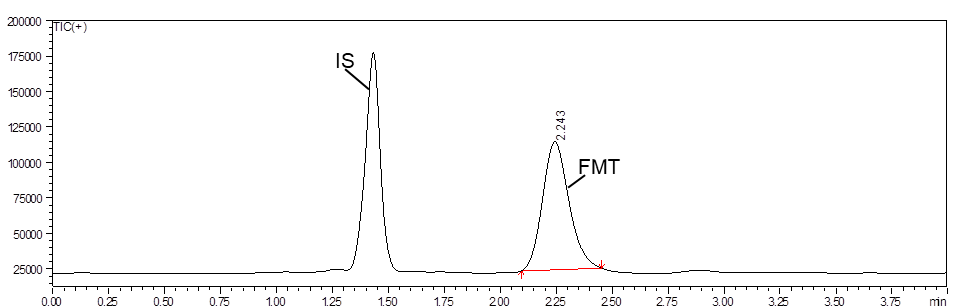


Figure S2. LC-MS chromatograms of IS (Irbesardan) and Standard (FMT)


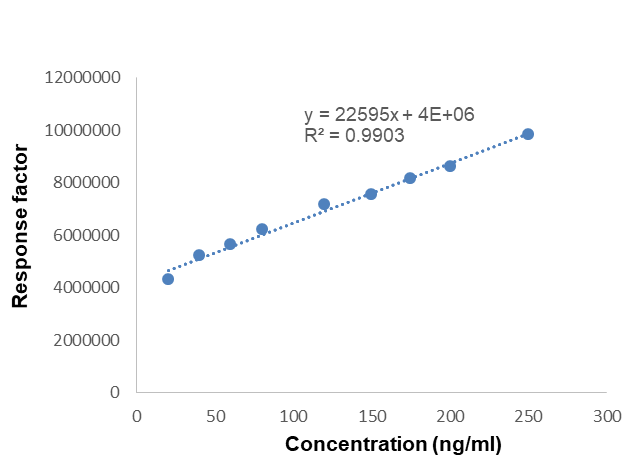


**Figure S3. Linearity plot for Naïve FMT.**

Table S1. Optimized LC-MS conditions for FMT and IBT (IS)

| **Analyte** | **Molecular weight (g/mol)** | **Ion mode** | **Precursor ion (m/z)** | **Dwell time (m/sec)** | **Retention time (min)** |
| --- | --- | --- | --- | --- | --- |
| FMT | 344.4 | Positive | 345.20 | 100 | 2.2 |
| IBT (IS) | 428.53 | Positive | 429.55 | 100 | 1.5 |

Preparation of standard and quality control solutions

Stock solution of FMT as well as IS (IBT) was prepared in methanol at the concentration of 1 mg/ml. The working standard solutions of FMT for calibration curves (20, 40, 60, 80, 120, 150, 175, 200 and 250 ng/ml) were prepared by diluting the stock solution with methanol. The quality control samples such as low-quality control (LQC), medium quality control (MQC) and high-quality control (HQC) were prepared at concentrations of 20, 120 and 225 ng/ml respectively.

Extraction of FMT from plasma and tissue samples

Protein precipitation method was used for extraction of FMT from plasma and tissue homogenate. In 2ml Eppendorf tube, 200μL of plasma or tissue homogenate, 200μL of IS was added. The volume was made up to 2ml with methanol. The tube was vortexed for 20 seconds and was centrifuged at 2000 rpm for 15 minutes. After centrifugation, supernatant was separated for LC-MS analysis ^1,2^.

Method validation

The developed LC-MS method was validated following the USFDA guidelines for bio-analytical method validation. The selectivity, accuracy, precision and recovery were taken into account for validation of the developed method using established protocols ^3^.

Table S2 shows that the developed method is accurate and can be reproduced. The stability test of plasma and tissue homogenate performed in six replicates under different storage conditions at four QC levels (10, 20, 120, 225 ng/mL) show results within the permissible limits (± 20% RSD) (Table S3).

| **Biological Samples** | **Analyte Conc(ng/mL)** | **Mean Conc** | **% Recovery** | **Absolute matrix effect** | **Intra-day** | | **Inter-day** | |
| --- | --- | --- | --- | --- | --- | --- | --- | --- |
|  |  |  |  |  | **Accuracy (%)** | **Precision (%RSD)** | **Accuracy (%)** | **Precision (%RSD)** |
| Plasma | 10 | 9.6 ± 0.7 | 96 | 0.94 | 95.88 | 7.29 | 94.32 | 8.12 |
|  | 20 | 19.45 ± 1.28 | 97.25 | 0.96 | 96.84 | 6.58 | 95.92 | 7.29 |
|  | 120 | 117.28 ± 6.85 | 97.73 | 0.98 | 97.21 | 5.84 | 96.12 | 6.31 |
|  | 225 | 220.58 ± 10.29 | 98.03 | 1.04 | 97.98 | 4.66 | 96.74 | 4.98 |
| Brain | 10 | 9.54 ± 0.8 | 95.4 | 0.94 | 95.07 | 8.38 | 94.15 | 9.11 |
|  | 20 | 19.25 ± 1.65 | 96.25 | 0.95 | 95.93 | 8.57 | 94.87 | 9.38 |
|  | 120 | 116.45 ± 7.85 | 97.04 | 0.97 | 96.72 | 6.74 | 95.36 | 7.42 |
|  | 225 | 219.51 ± 10.99 | 97.56 | 1.09 | 97.13 | 5.01 | 96.48 | 5.76 |
| Heart | 10 | 9.42 ± 0.85 | 94.2 | 0.92 | 94.02 | 9.02 | 93.15 | 9.75 |
|  | 20 | 19.12 ± 1.45 | 95.6 | 0.96 | 95.14 | 7.58 | 94.64 | 8.12 |
|  | 120 | 116.2 ± 7.42 | 96.8 | 0.98 | 96.39 | 6.38 | 95.72 | 7.02 |
|  | 225 | 218.12 ± 10.85 | 96.9 | 1.13 | 96.51 | 4.97 | 95.83 | 5.32 |
| Lungs | 10 | 9.14 ± 0.9 | 91.4 | 0.95 | 91.03 | 9.84 | 90.08 | 10.19 |
|  | 20 | 18.57 ± 2.11 | 92.85 | 0.96 | 92.42 | 11.36 | 91.42 | 11.85 |
|  | 120 | 112.72 ± 8.48 | 93.94 | 0.98 | 93.16 | 7.52 | 92.32 | 8.16 |
|  | 225 | 215.29 ± 11.52 | 95.68 | 1.15 | 94.91 | 5.35 | 93.12 | 6.11 |

Table S2. Percentage recovery, absolute matrix effect, intra and inter-day accuracy and precision analysis of FMT in rat plasma and tissues

The data represents mean ± SD, n = 6.

Table S3. Stability study of FMT in rat plasma and tissues.

| **Biological Samples** | **Analyte Conc(ng/mL)** | **Freeze Thaw(3 cycles at -70 ±2°C)** | | **Short term (25°C for 24 h)** | | **Stock Solution (25°C for 24 h)** | |
| --- | --- | --- | --- | --- | --- | --- | --- |
|  |  | **Accuracy (%)** | **Precision (%RSD)** | **Accuracy (%)** | **Precision (%RSD)** | **Accuracy (%)** | **Precision (%RSD)** |
| Plasma | 10 | 95.88 | 7.29 | 94.78 | 7.54 | 97.54 | 5.01 |
|  | 20 | 96.84 | 6.58 | 95.12 | 7.01 | 97.98 | 4.56 |
|  | 120 | 97.21 | 5.84 | 95.75 | 6.28 | 98.35 | 3.97 |
|  | 225 | 97.98 | 4.66 | 96.42 | 5.37 | 98.76 | 3.32 |
| Brain | 10 | 95.07 | 8.38 | 94.06 | 9.12 | 96.12 | 6.42 |
|  | 20 | 95.93 | 8.57 | 94.85 | 9.48 | 97.54 | 6.11 |
|  | 120 | 96.72 | 6.74 | 95.23 | 7.29 | 97.88 | 4.89 |
|  | 225 | 97.13 | 5.01 | 96.11 | 5.94 | 98.62 | 4.08 |
| Heart | 10 | 94.02 | 9.02 | 92.75 | 10.54 | 96.54 | 5.35 |
|  | 20 | 95.14 | 7.58 | 93.59 | 8.03 | 97.12 | 4.98 |
|  | 120 | 96.39 | 6.38 | 95.34 | 7.12 | 97.35 | 4.09 |
|  | 225 | 96.51 | 4.97 | 95.55 | 6.03 | 98.03 | 3.21 |
| Lungs | 10 | 91.03 | 9.84 | 90.22 | 10.97 | 96.45 | 5.87 |
|  | 20 | 92.42 | 11.36 | 91.48 | 12.69 | 97.04 | 5.62 |
|  | 120 | 93.16 | 7.52 | 91.87 | 8.21 | 97.98 | 4.12 |
|  | 225 | 94.91 | 5.35 | 93.26 | 6.84 | 98.48 | 3.59 |

S1.2 Pre-formulation studies

Compatibility studies

The DSC thermogram of FMT shows an endothermic peak at 142.03°C whereas SA shows at 53.28 °C. Their physical mixture shows no changes in the endothermic peaks as compared to individual agents, indicating thermal stress stability between the two mixtures (Figure S4).


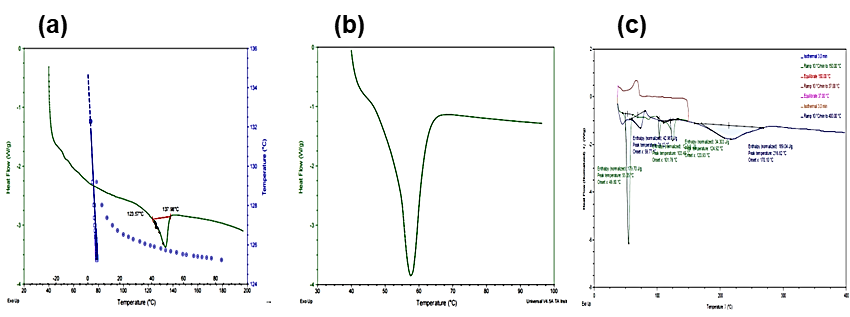


Figure S4. DSC of (a) FMT, (b) SA and (c) Physical mixture (FMT+SA)

FT-IR spectrum of FMT showed characteristic peaks at 3481.63 cm^-1^ (OH), 3121.89 cm^-1^ (aromatic C-H), 3043.77 cm^-1^ (N-H), 2848.96 cm^-1^ (aliphatic C-H), 1689.70 cm^-1^ (aldehyde O=C-H) and 1375.29 cm^-1^ (aromatic C=C). The spectrum of stearylamine (SA) showed characteristic peaks at 3332.14 cm^-1^(NH_2_ primary amine), 2849.92 cm^-1^ (C-H), 1471.74 cm^-1^ (CH_2_) and 1243.16 (CN). The physical mixture (FMT+SA) spectra exhibited the same characteristics as individual compounds without any presence of new or absence of the characteristic peaks (Figure S5). Thus, these results support the molecular and chemical compatibility between FMT and SA.


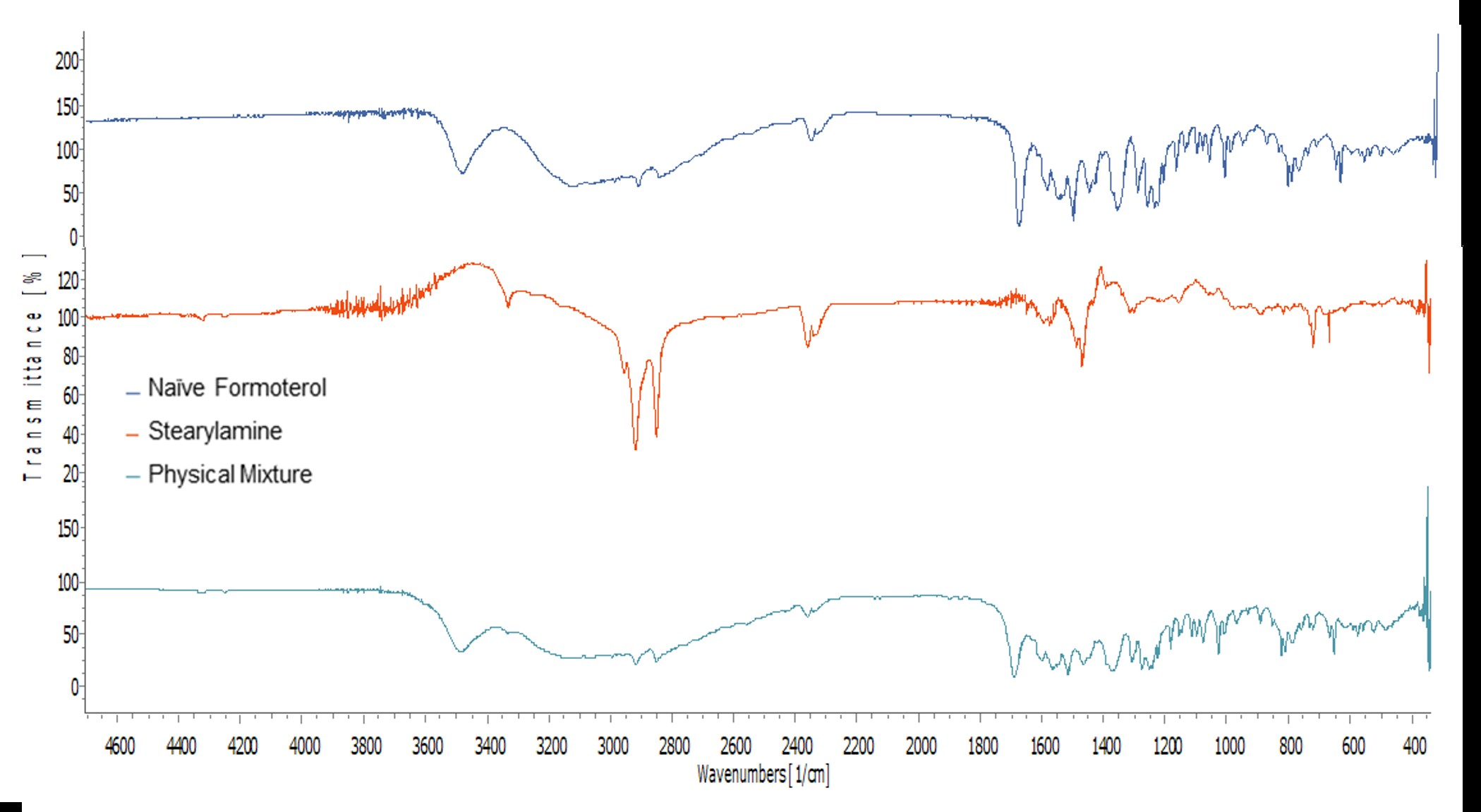


**Figure S5. FT-IR spectra of FMT, SA, and physical mixtures (FMT+SA)**


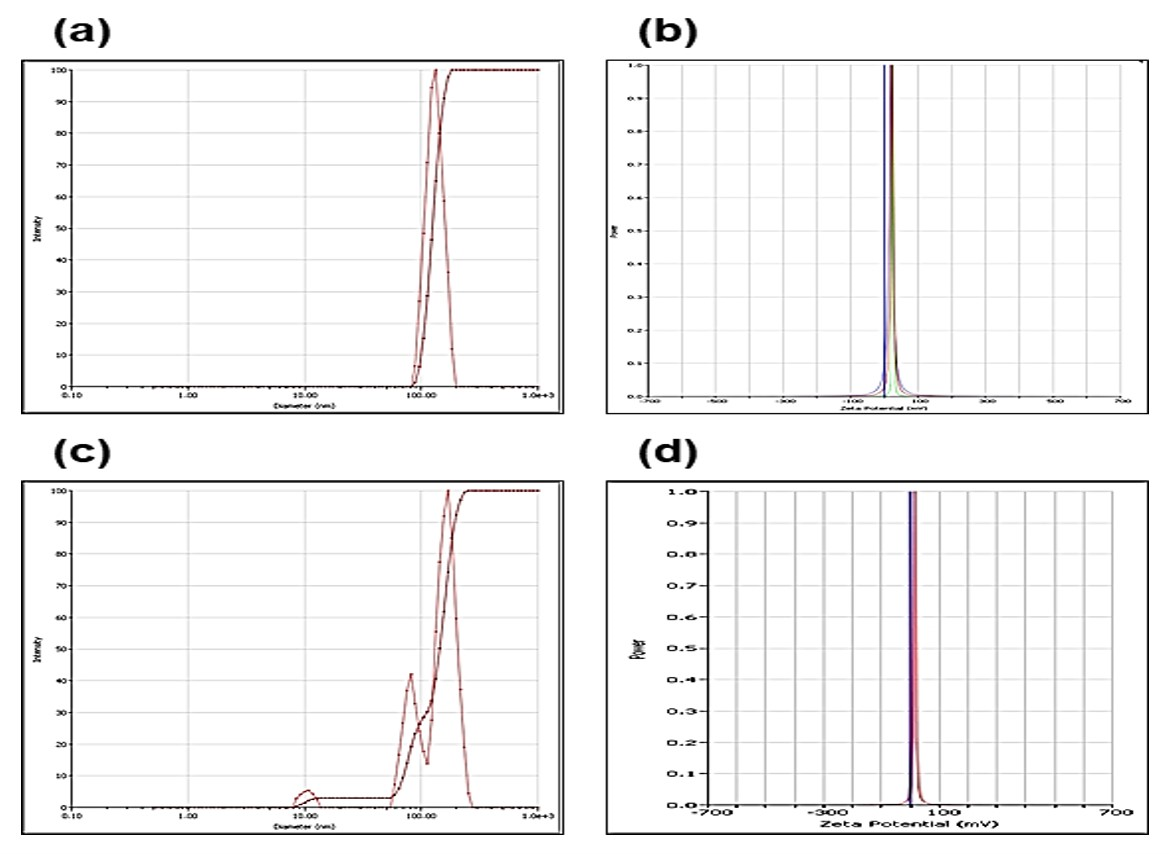


Figure S6. (a) PS (nm) of FMT-SLNs-PS80 (trial batch), (b) ZP (mV) of FMT-SLNs-PS80 (trial batch), (c) PS (nm) of Blank-SLNs and (c) ZP (mV) of Blank-SLNs.

Table S4. Responses for dependent variables for 15 experimental batches

| **Std**  **order** | **Run**  **Order** | **Surfactant**  **Concentration**  **% w/v** | **Sonication**  **Amplitude**  **(%)** | **Sonication**  **Time**  **(min)** | **PS**  **Nm** | **ZP**  **mV** |
| --- | --- | --- | --- | --- | --- | --- |
| 13 | 1 | 4 | 80 | 6 | 168.99±2.06 | 23.94±0.21 |
| 5 | 2 | 2 | 80 | 3 | 201.42±2.53 | 23.93±0.45 |
| 9 | 3 | 4 | 60 | 3 | 186.49±2.13 | 24.12±0.75 |
| 14 | 4 | 4 | 80 | 6 | 168.95±1.59 | 23.92±0.41 |
| 2 | 5 | 6 | 60 | 6 | 199.24±3.11 | 20.85±0.27 |
| 6 | 6 | 6 | 80 | 3 | 186.51±1.38 | 23.09±0.63 |
| 3 | 7 | 2 | 100 | 6 | 219.32±2.33 | 20.02±1.09 |
| 4 | 8 | 6 | 100 | 6 | 208.45±1.66 | 20.67±0.72 |
| 8 | 9 | 6 | 80 | 9 | 174.91±1.49 | 23.93±0.61 |
| 11 | 10 | 4 | 60 | 9 | 170.05±2.62 | 22.67±1.12 |
| .21 | 11 | 2 | 60 | 6 | 196.63±1.35 | 21.27±0.43 |
| 7 | 12 | 2 | 80 | 9 | 169.12±2.41 | 21.64±0.10 |
| 15 | 13 | 4 | 80 | 6 | 157.47±1.89 | 23.74±0.68 |
| 10 | 14 | 4 | 100 | 3 | 203.47±2.50 | 22.93±0.36 |
| 12 | 15 | 4 | 100 | 9 | 179.64±1.95 | 22.79±0.55 |

Values are mean±S.D, (n=3)


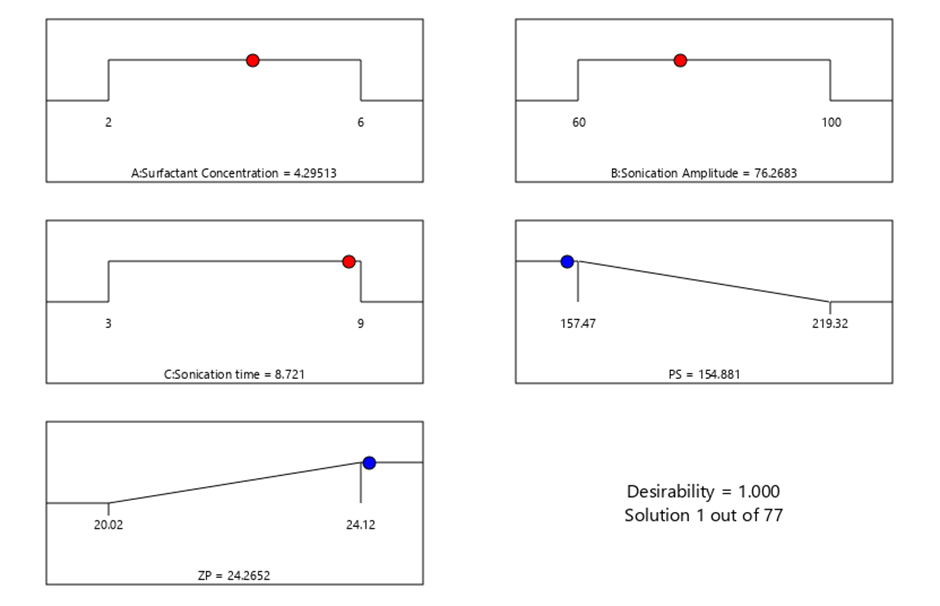


Figure S7. Software predicted surfactant concentration, sonication amplitude, and sonication time for preparing optimised batch

S1.2.1 Effect of surfactant concentration, sonication amplitude, and sonication time on PS

Table S5 shows the ANOVA findings for PS of FMT-SLNs-PS80 with an F-value of 26.45 and an R^2^ of 0.9794. The PS model produced by the software was noteworthy. ANOVA was used to determine the influence of each linear, quadratic, and interaction component, with the significance represented by the P-value (p<0.05) (Table S5). For this PS model, the polynomial equation derived is,

**PS(nm) =** 165.14-2.17 A + 7.31 B - 10.52C-3.37 AB + 5.18 AC - 1.85 BC + 19.43 A^2^ + 21.35 B^2^ - 1.57 C^2.^

Where,

A is surfactant concentration, sonication amplitude B, and sonication time C. They have substantial influence on PS (p<0.05).

The positive sign in the above regression equation signifies synergistic effects, while the negative sign indicates antagonistic effects. Figure S8 shows the major effect plots of surfactant concentration, sonication amplitude, and sonication time on the PS of FMT-SLNs-PS80.

Table S5. ANOVA data of response surface quadratic model for PS of FMT-SLNs-PS80

| **Source** | **Sum of Squares** | **Df** | **Mean Square** | **F-value** | **p-value** |  |
| --- | --- | --- | --- | --- | --- | --- |
| **Model** | 4449.29 | 9 | 494.37 | 26.45 | 0.0011 | Significant |
| A-Surfactant Concentration | 37.76 | 1 | 37.76 | 2.02 | 0.2145 |  |
| B-Sonication Amplitude | 427.34 | 1 | 427.34 | 22.86 | 0.0050 |  |
| C-Sonication time | 885.57 | 1 | 885.57 | 47.38 | 0.0010 |  |
| AB | 45.43 | 1 | 45.43 | 2.43 | 0.1797 |  |
| AC | 107.12 | 1 | 107.12 | 5.73 | 0.0621 |  |
| BC | 13.65 | 1 | 13.65 | 0.7304 | 0.4318 |  |
| A² | 1393.28 | 1 | 1393.28 | 74.54 | 0.0003 |  |
| B² | 1682.71 | 1 | 1682.71 | 90.02 | 0.0002 |  |
| C² | 9.13 | 1 | 9.13 | 0.4882 | 0.5159 |  |
| **Residual** | 93.46 | 5 | 18.69 |  |  |  |
| Lack of Fit | 5.29 | 3 | 1.76 | 0.0400 | 0.9865 | not significant |
| Pure Error | 88.17 | 2 | 44.08 |  |  |  |
| **Cor Total** | 4542.75 | 14 |  |  |  |  |

**
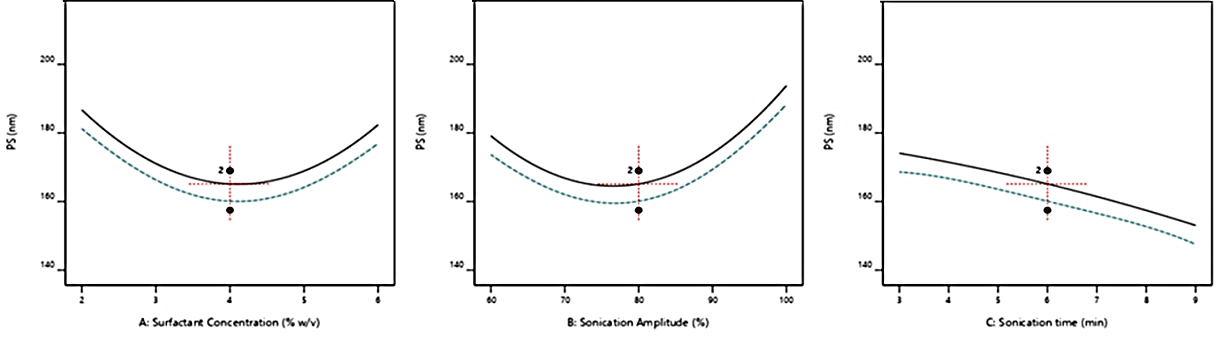
Regression Analysis**

|  | **R²** | 0.9794 |
| --- | --- | --- |
|  | **Adjusted R²** | 0.9424 |
|  | **Predicted R²** | 0.9377 |
|  | **Adeq Precision** | 15.1910 |

**Figure S8. Main effect plots of surfactant concentration, sonication amplitude and sonication time on PS of FMT-SLNs-PS80**.

S1.2.2 Effect of independent variables on ZP

Table S6 shows the ANOVA findings for ZP of FMT-SLNs-PS80 with F-value of 65.42 and R^2^ value of 99.82%. The ZP model generated by the software was significant. ANOVA was used to determine the influence of each linear, quadratic, and interaction term, with the significance of the terms represented by the P-value (p<0.05). For this model of ZP, the polynomial equation derived is

**ZP (mV)=** +23.87 + 0.2100 A - 0.3125 B - 0.3800 C + 0.2675 AB + 0.7825 AC + 0.3275 BC - 1.57 A² - 1.59 B²+ 0.8529 C²

Where,

A is surfactant concentration, sonication amplitude B, and sonication time C. They have substantial influence on ZP (p<0.05).

The main effect plots of A, B and C on ZP of FMT-SLNs-PS80 is shown in Figure S9.

Table S6. ANOVA data of response surface quadratic model for ZP of FMT-SLNs-PS80

| **Source** | **Sum of Squares** | **df** | **Mean Square** | **F-value** | **p-value** |  |
| --- | --- | --- | --- | --- | --- | --- |
| **Model** | 26.96 | 9 | 3.00 | 65.42 | 0.0001 | Significant |
| **A-Surfactant Concentration** | 0.3528 | 1 | 0.3528 | 7.70 | 0.0391 |  |
| **B-Sonication Amplitude** | 0.7813 | 1 | 0.7813 | 17.06 | 0.0091 |  |
| **C-Sonication time** | 1.16 | 1 | 1.16 | 25.23 | 0.0040 |  |
| **AB** | 0.2862 | 1 | 0.2862 | 6.25 | 0.0545 |  |
| **AC** | 2.45 | 1 | 2.45 | 53.48 | 0.0007 |  |
| **BC** | 0.4290 | 1 | 0.4290 | 9.37 | 0.0281 |  |
| **A²** | 9.13 | 1 | 9.13 | 199.27 | < 0.0001 |  |
| **B²** | 9.36 | 1 | 9.36 | 204.37 | < 0.0001 |  |
| **C²** | 2.69 | 1 | 2.69 | 58.66 | 0.0006 |  |
| **Residual** | 0.2290 | 5 | 0.0458 |  |  |  |
| **Lack of Fit** | 0.2047 | 3 | 0.0682 | 5.62 | 0.1547 | not significant |
| **Pure Error** | 0.0243 | 2 | 0.0121 |  |  |  |
| **Cor Total** | 27.19 | 14 |  |  |  |  |

**Regression Analysis**

|  | **R²** | 0.9916 |
| --- | --- | --- |
|  | **Adjusted R²** | 0.9764 |
|  | **Predicted R²** | 0.8775 |
|  | **Adeq Precision** | 24.2381 |

**
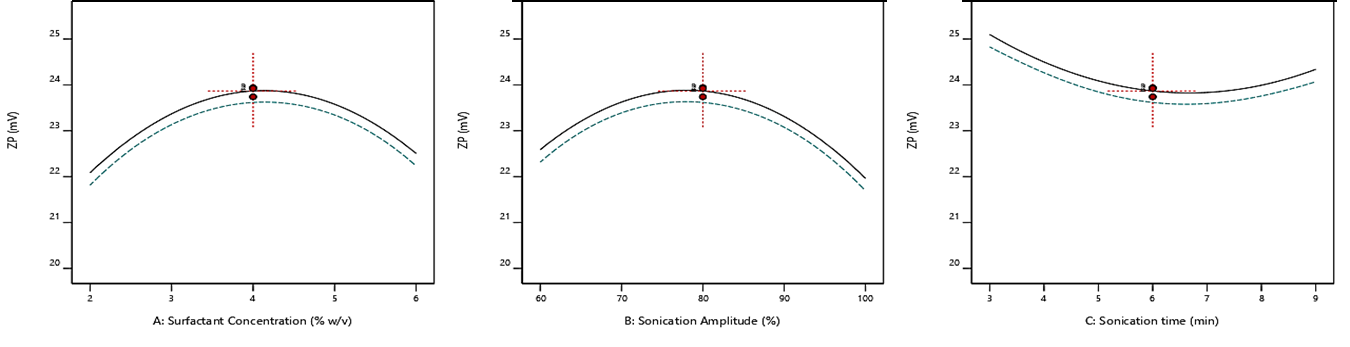
**

Figure S9. Main effect Plots of surfactant concentration, sonication amplitude and sonication time on ZP of FMT-SLNs-PS80.


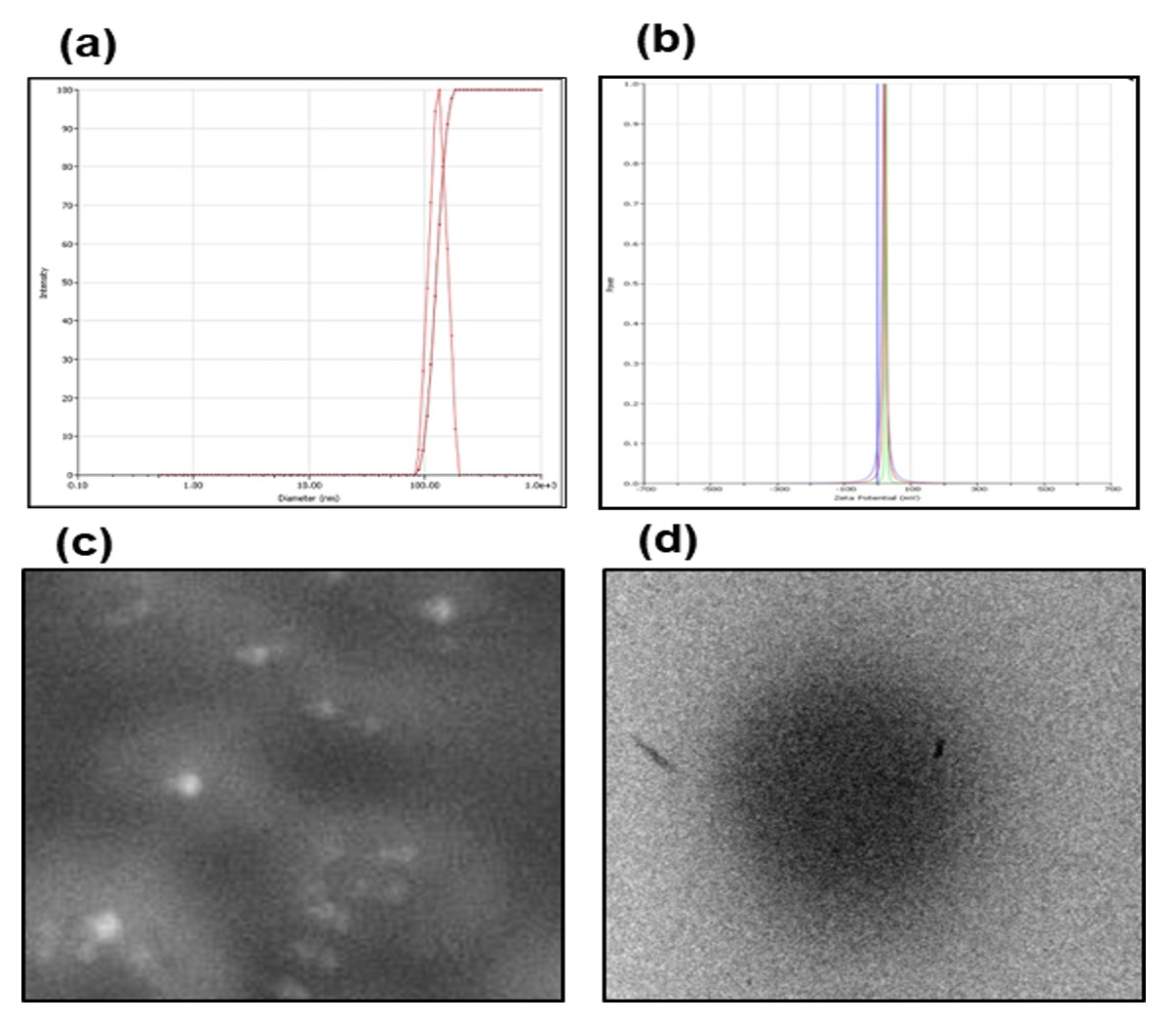


Figure S10. Characterization of optimized batch of FMT-SLNs-PS80: (a) PS (nm), and (b) ZP (mV), (c) SEM image, and (d) TEM image.

S1.3. Lyophilization of FMT-SLNs-PS80

Table S7 shows the results where slight increase in the PS, ZP and PDI were observed.

Table S7. PS, ZP and PDI of FMT-SLNs-PS80 before and after lyophilization

|  | **Before lyophilisation** | **After lyophilisation** |
| --- | --- | --- |
| PS (nm) | 151.28±0.33 | 154.81±7.92 |
| ZP (mV) | 22.11±3.18 | 22.17±2.09 |
| PDI (%) | 0.143±0.11 | 0.264±0.01 |

Values are mean ± S.D(n=3)

S1.4 Stability studies

Table S8 summarises the findings. When compared to FMT-SLNs-PS80 suspension, freeze dried FMT-SLNs-PS80 exhibited higher stability with respect to PS, ZP, and PDI for up to 6 months. At all temperatures and humidity levels, modest drug content changes were observed. The findings show that the freeze-dried product is stable for 6 months when kept refrigerated.

Table S8. Stability result for FMT-SLNs-PS80 suspension and lyophilized formulation

| **Sampling time** | **FMT-SLNs-PS80 Suspension** | | | **Freeze dried FMT-SLNs-PS80** | | |
| --- | --- | --- | --- | --- | --- | --- |
|  | **5±3^o^C** | **25±2^o^C/65± 5% RH** | **40±2^o^C/75±5% RH** | **5±3^o^C** | **25±2^o^C/65±5%RH** | **40±2^o^C/75±5% RH** |
| **Particle size(nm)** | | | | | | |
| **Initial** | 151.28±0.33 | 151.28±0.33 | 151.28±0.33 | 154.81±7.92 | 154.81±7.92 | 154.81±7.92 |
| **1 month** | 154.12±0.21 | 155.08±0.12 | 167.02±0.31 | 156.07±0.13 | 155.24±1.52 | 156.32±2.17 |
| **3 months** | 156.34±0.61 | 160.14±0.21 | 186.27±0.11 | 159.12±0.31 | 156.32±2.01 | 159.64±2.03 |
| **6 months** | 158.71±1.03 | 165.19±0.19 | 205.11±0.93 | 163.09±0.57 | 156.11±1.19 | 160.17±3.01 |
| **Zeta potential (mV)** | | | | | | |
| **Initial** | 22.11±3.18 | 22.11±3.18 | 22.11±3.18 | 22.17±2.09 | 22.17±2.09 | 22.17±2.09 |
| **1 month** | 23.08±0.13 | 23.14±2.17 | 23.07±1.17 | 22.63±0.18 | 22.73±2.19 | 22.97±3.71 |
| **3 months** | 23.19±1.25 | 23.64±2.91 | 23.66±0.19 | 23.02±3.12 | 23.08±1.59 | 23.32±1.02 |
| **6 months** | 23.39±1.41 | 24.02±1.25 | 25.09±0.32 | 24.37±2.50 | 23.79±1.25 | 23.85±1.29 |
| **Polydispersity Index** | | | | | | |
| **Initial** | 0.143±0.11 | 0.143±0.11 | 0.143±0.11 | 0.264±0.01 | 0.264±0.01 | 0.264±0.01 |
| **1 month** | 0.150±0.31 | 0.160±0.01 | 0.179±0.32 | 0.265±0.19 | 0.267±0.14 | 0.269±0.05 |
| **3 months** | 0.165±0.01 | 0.172±0.07 | 0.207±0.18 | 0.265±2.14 | 0.269±2.06 | 0.271±0.12 |
| **6 months** | 0.171±0.12 | 0.180±0.15 | 0.211±0.22 | 0.267±1.56 | 0.272±2.16 | 0.375±0.08 |
| **Drug Content (%)** | | | | | | |
| **6 months** | 98.05±1.39 | 96.35±1.99 | 96.09±2.39 | 98.41±1.31 | 96.91±1.67 | 98.11±1.89 |
| The values are mean ± S.D (n=3) | | | | | | |

Table S9*. In-vitro* release data of FMT-SLNs-PS80 and Naïve FMT

| **Time (h)** | **Percentage drug release (%)** | |
| --- | --- | --- |
|  | **FMT-SLNs-PS80** | **Naïve FMT** |
| 0 | 0 | 0 |
| 0.5 | 9.78±0.87 | 15.42±2.06 |
| 1 | 10.08±1.94 | 33.54±2.14 |
| 2 | 14.63±2.49 | 41.17±2.11 |
| 4 | 20.07±2.16 | 58.97±2.39 |
| 6 | 39.29±2.30 | 72.06±1.96 |
| 8 | 44.42±2.55 | 84.64±2.35 |
| 10 | 49.53±2.39 | 96.54±1.99 |
| 12 | 59.58±2.43 |  |
| 24 | 74.32±2.68 |  |
| 48 | 90.42±3.07 |  |

The values are mean ± S.D., (n=3)

**Figure S11. Zero-order plot of FMT-SLNs-PS80**.

**Figure** S12. Higuchi Plot of FMT-SLNs-PS80

Figure S13. Korsmeyer-Peppas plot of FMT-SLNs-PS80

Figure S14. First-order plot of FMT-SLNs-PS80

Figure S15. Hixson Crowell plot of FMT-SLNs-PS80

Table. S10. Standard for ROS

| **Sample** | **OD @ 450nm** | **ROS (ng/ml)** |
| --- | --- | --- |
| Standard1 | 0.8495 | 25 |
| Standard2 | 0.4025 | 13 |
| Standard3 | 0.2246 | 6.5 |
| Standard4 | 0.1047 | 3.3 |
| Standard5 | 0.0552 | 1.5 |

Figure S16. Standard curve of ROS

Table S11. Standard for Super Oxidase Dismutase (SOD)

| **Sample** | **OD @ 450nm** | | **SOD (U/L)** |
| --- | --- | --- | --- |
| Standard1 | 1.4598 | 100 | |
| Standard2 | 0.7911 | 50 | |
| Standard3 | 0.391 | 25 | |
| Standard4 | 0.1623 | 12.5 | |
| Standard5 | 0.1166 | 6.25 | |
| Standard6 | 0.0572 | 3.2 | |

Figure S17. Standard curve of Super Oxidase Dismutase (U/L)

Table S12. Standard for Catalase (CAT)

| **Sample** | **OD @ 450nm** | **CAT (U/L)** |
| --- | --- | --- |
| Standard1 | 1.3263 | 5 |
| Standard2 | 0.7893 | 2.5 |
| Standard3 | 0.4222 | 1.3 |
| Standard4 | 0.2284 | 0.52 |
| Standard5 | 0.1346 | 0.3 |

Figure S18. Standard curve of Catalase (U/L)


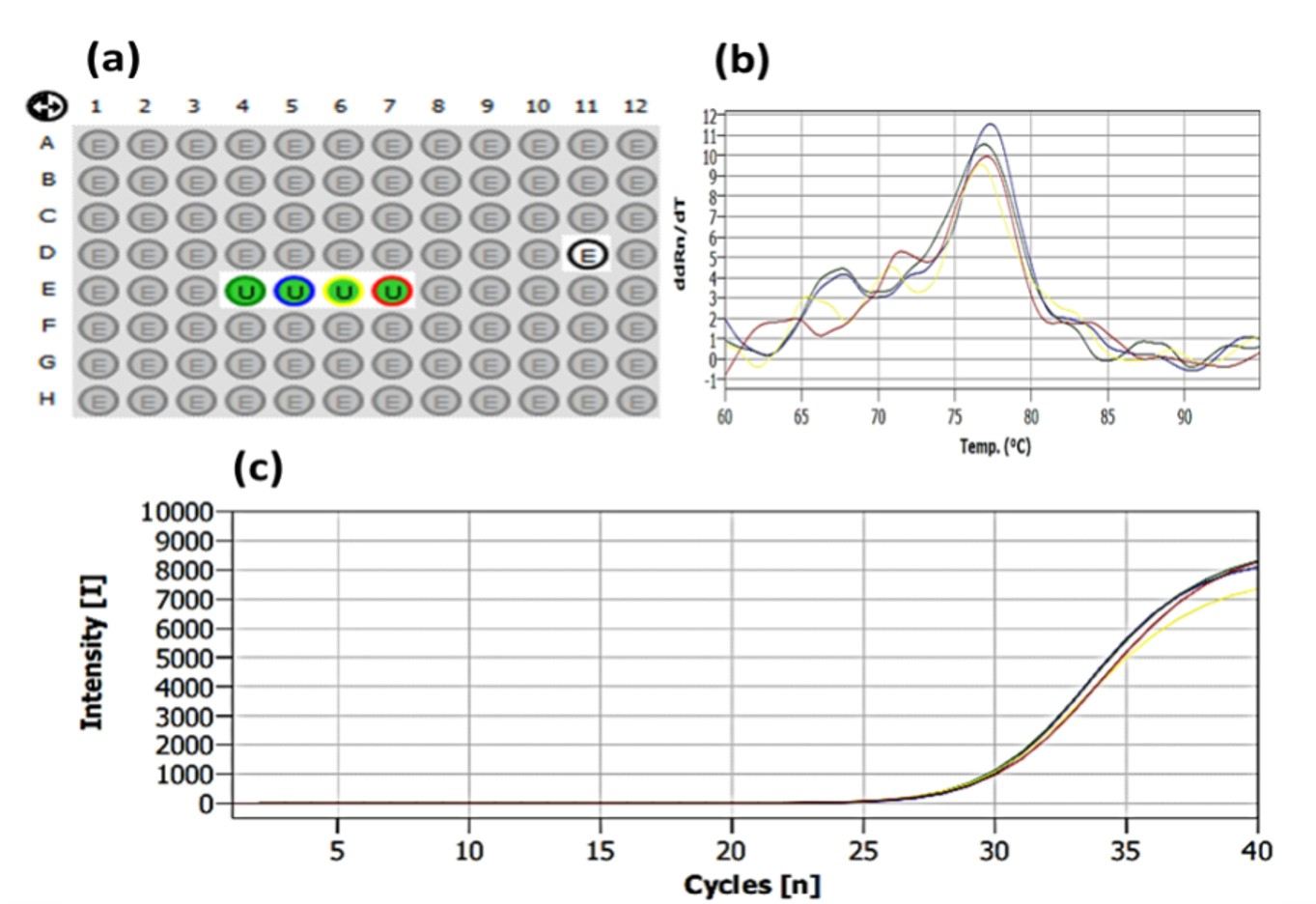


Figure S19 (a) Sampling in 96 well plate, (b) Melting curves and (c) Amplification cycle for *in-vitro* SNCA gene expression study

Table S13. Calculation of ∆Ct, ∆∆Ct and 2^-(∆∆Ct)

|  |  |  |  |  |  |  |
| --- | --- | --- | --- | --- | --- | --- |
| **Group** | **Treatment** | **House keeping gene** | **SNCA** | **∆Ct** | **∆∆Ct** | **2^^-(∆∆Ct)^** |
| **UT** | **Untreated** | 24.21 | 25.83 | 1.62 | 0.0 | 1.00 |
| **S1** | **Rotenone (10 μM)** | 25.29 | 26.04 | 0.75 | -0.9 | 1.83 |
| **S2** | **Rotenone+Naïve FMT (12.5 μg/ml)** | 24.55 | 25.77 | 1.22 | -0.4 | 1.32 |
| **S3** | **Rotenone+ FMT-SLNs-PS80 (69.25μg/ml)** | 24.73 | 26.28 | 1.55 | -0.1 | 1.05 |

**Table S14. Locomotor activity in PD animal model**

The values are mean ± SD value (n=6).

| **Days** | **Normal control** | **Disease control** | **Naïve FMT (5mg/kg, p.o)** | **Blank SLNs**  **(5mg/kg, p.o)** | **FMT-SLNs-PS80 (10mg/kg, p.o)** | **FMT-SLNs-PS80 (50mg/kg, p.o)** | **FMT-SLNs-PS80 (100mg/kg, p.o)** |  |
| --- | --- | --- | --- | --- | --- | --- | --- | --- |
| **0** | 482.38±3.02 | 482.13±3.64 | 481.34±3.24 | 482.85±2.42 | 482.50±3.34 | 483.09±3.25 | 482.18 ±3.11 | |
| **7** | 484.5±2.88 | 470.5±2.83 | 486.12±3.46 | 468.2±2.68 | 485.63±3.74 | 495.12±3.31 | 503.62 ±2.77 | |
| **14** | 485.75±2.96 | 406.5±2.67 | 489.24±2.98 | 414.25±2.62 | 490.25±2.6 | 497.25±2.6 | 511.13 ±2.7 | |
| **21** | 487.88±3.09 | 363.75±2.86 | 490.12±2.64 | 359.06±2.91 | 495.27±2.38 | 500.12±2.23 | 519.12 ±2.1 | |

Table S15. **Muscle grip strength test in PD animal model**

| **Days** | **Normal control** | **Disease control** | **Naïve FMT**  **(5mg/kg, p.o)** | **Blank SLNs (5mg/kg, p.o)** | **FMT-SLNs-PS80 (10mg/kg, p.o)** | **FMT-SLNs-PS80 (50mg/kg, p.o)** | **FMT-SLNs-PS80 (100mg/kg, p.o)** |
| --- | --- | --- | --- | --- | --- | --- | --- |
| 0 | 158.12 ±1.55 | 159.25 ± 2.12 | 158.98 ± 1.58 | 158.24 ± 1.98 | 158.50 ± 2.77 | 159.87 ±2.03 | 159.25 ±2.31 |
| 7 | 161.87 ±1.45 | 141.75 ± 2.60 | 159.12 ± 2.64 | 139.23 ± 2.54 | 161.87 ± 2.53 | 166.72 ± 1.72 | 172.12 ± 2.47 |
| 14 | 165.75 ±1.28 | 73.5 ± 2.61 | 163.21 ± 2.43 | 72.12 ± 2.34 | 177.25 ± 2.37 | 180.83 ± 2.16 | 189.37 ± 2.06 |
| 21 | 171.62 ±2.5 | 52.37 ± 4.83 | 162.24 ± 2.82 | 59.09 ± 2.87 | 182.87 ±1.64 | 192.12 ± 1.12 | 206.62 ± 2.92 |

The values are mean ± SD value (n=6).

Table S16. **Memory function test in PD animal model**

| **Days** | **Normal control** | **Disease control** | **Naïve FMT (5mg/kg, p.o)** | **Blank SLNs**  **(5mg/kg, p.o)** | **FMT-SLNs-PS80 (10mg/kg, p.o)** | **FMT-SLNs-PS80 (50mg/kg, p.o)** | **FMT-SLNs-PS80 (100mg/kg, p.o)** |
| --- | --- | --- | --- | --- | --- | --- | --- |
| 0 | 65.46±1.50 | 65.23±2.29 | 65.42 ±1.52 | 65.25±2.24 | 65.76±1.62 | 65.71±1.9 | 65.72±1.90 |
| 7 | 69.33±1.95 | 59.85±1.25 | 67.12 ±1.87 | 61.54±1.98 | 71.57±1.69 | 72.18±1.09 | 73.52±1.06 |
| 14 | 71.95±2.30 | 56.18±2.72 | 69.24 ±2.54 | 60.21±1.76 | 76.95±1.94 | 79.31±1.06 | 82.92±1.49 |
| 21 | 75.58±2.96 | 39.52±7.6 | 73.12 ±1.89 | 41.98±1.56 | 83.71±2.03 | 85.65±1.48 | 85.39±1.50 |

The values are mean ± SD value (n=6).


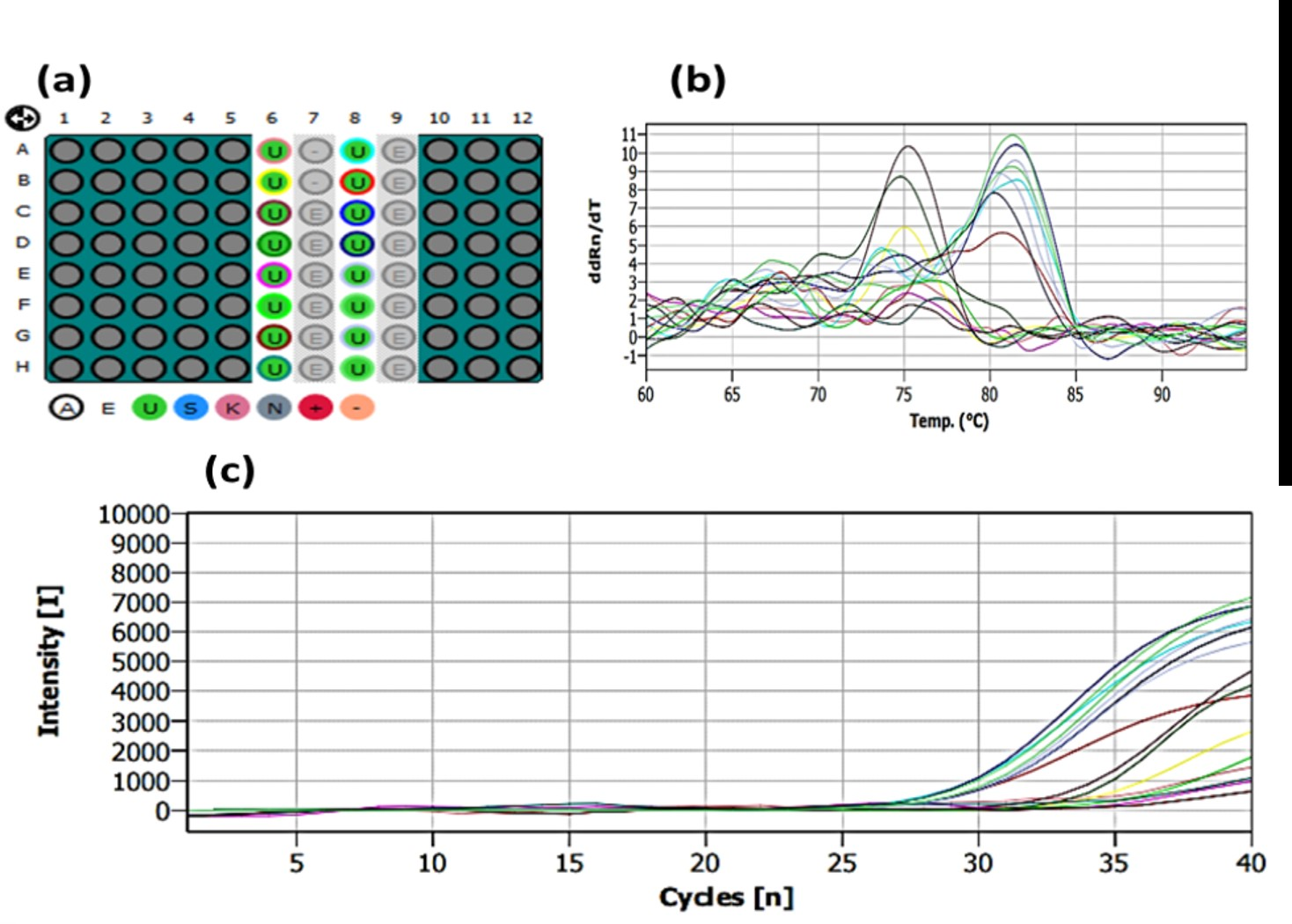


Figure S20. (a) Sampling in 96 well plate, (b) Melting curves and (c) Amplification cycle for *in-vivo* SNCA gene expression study

Table S17. Calculation of ∆Ct, ∆∆Ct and 2^^-(∆∆Ct)^

| **Group** | **Treatment** | **House keeping gene** | **SNCA** | **∆Ct** | **∆∆Ct** | **2^^-(∆∆Ct)^** |
| --- | --- | --- | --- | --- | --- | --- |
| **S1** | Normal Control | 24.21 | 24.38 | 0.17 | 0.0 | 1.00 |
| **S2** | Disease Control | 24.73 | 24.8 | 0.07 | -0.1 | 1.07 |
| **S3** | Blank SLNs  (5mg/kg; p.o.) | 24.55 | 24.91 | 0.36 | 0.2 | 0.88 |
| **S4** | Naive FMT  (5mg/kg; p.o.) | 24.52 | 25.04 | 0.52 | 0.4 | 0.78 |
| **S5** | FMT-SLNs-PS80  (10mg/kg; p.o.) | 24.76 | 25.54 | 0.78 | 0.6 | 0.66 |
| **S6** | FMT-SLNs-PS80  (50mg/kg; p.o.) | 24.82 | 26.14 | 1.32 | 1.2 | 0.45 |
| **S7** | FMT-SLNs-PS80  (100mg/kg; p.o**.**) | 24.93 | 26.56 | 1.63 | 1.5 | 0.36 |

S2. Materials and Methods

S2.1 Surface morphology of FMT-SLNs-PS80

SEM analysis

The accelerating voltage of 20kV was used in the SEM investigation. The FMT-SLNs P80-NPs suspension (4-8 mg) was evenly placed on aluminium stubs and allowed to settle and surplus liquid was blotted using paper. A small layer of gold (150 A°) was sprayed onto the particles using a sputter coater, making the sample conductive. Following that, the samples were inspected using a scanning electron microscope (SEM) ^4,5^.

TEM analysis

Negative staining was used in the TEM investigation, which was performed at 200 kV. The sample was deposited onto 200-mess copper grids coated with carbon after being diluted with double distilled water (1:100). A drop of phosphotungstic acid (2% w/v) to the samples was added to stain for 0.5 minutes, the excess stain was removed by paper bolting. The grid was air-dried at room temperature to form a thin film of the sample. The grids were examined using a TEM instrument ^4,6^.

S2.2 Lyophilization of optimized FMT-SLNs-PS80

To facilitate long term stability, the optimized batch of FMT-SLNs-PS80 was freeze dried. FMT-SLNs-PS80 suspension (25ml) was taken in tray and mannitol (5%w/v, cryoprotectant) was poured and stirred gently for 1-2 minutes. The tray was kept in deep freezer and the temperature was gradually decreased to -40°C at air pressure for 145 minutes at a rate of 1°C per minute. Finally, the tray was transferred to lyophilizer, where it was lyophilized for 24 hours at a temperature of -70°C.

S2.3 Stability Studies

Stability testing of the FMT-SLNs-PS80 suspension and lyophilized FMT-SLNs-PS80 was performed following the ICH Guidelines, ICH Q1 A (R2), at different temperatures and relative humidity (RH) for 6 months at 5±3°C, 25±2°C/65±5% RH and 40±2°C/75±5% RH. The PS, ZP and PDI of FMT-SLNs-PS80 were measured at 0, 1, 3, and 6 months. Drug content was examined at the end of 6 months ^7^.

S.2.4 Cytotoxicity studies

**Principle:** MTT assay is a colorimetric technique for determining cell proliferation and cytotoxicity. It depends on the reduction of the yellow tetrazolium dye 3-(4, 5-dimethyl-thiazol-2-yl)-2,5-diphenyl tetrazolium bromide (MTT) to formazan crystals. Mitochondrial lactate dehydrogenase converts MTT to insoluble formazan crystals, which when dissolved in a suitable solvent produce a purple colour whose intensity is proportional to the amount of live cells and can be measured spectrophotometrically at 570nm ^8-10^.

**Protocol:**

Cell suspension of 200μl was seeded in 96-well plate at 20,000 cells per well devoid of the test agent and allowed to grow in DMEM media for 24 hours. The cells were treated with different concentrations (12.5-200µg/ml) of blank SLNs, Naïve FMT and FMT-SLNs-PS80. As a positive control, doxorubicin 5µM/ml was used. The plate was incubated at 37°C in 5% CO_2_ atmosphere for 24 hours. The spent media was discarded from the plates. MTT reagent was added to the total volume concentration of 0.5mg/ml. To avoid light exposure, the plate was wrapped with aluminium foil and incubated for 3 hours. The MTT reagent was discarded and 100μl of dimethyl sulfoxide (DMSO, solubilisation solution) was added. To aid dissolution, a gyratory shaker was used to a gently swirl the mixture. Pipetting was done to completely dissolve the MTT formazan crystals especially the dense cultures. The absorbance was measured using an ELISA reader at 570nm.The percentage cell viability was computed using formula:

% cell viability= $\frac{Sample absorbance}{Control absorbance}x 100$

The CTC_50_ value was calculated using the linear regression equation,

Y= Mx+C.

where, Y = 50, and the values of M and C derived from the viability graph.

S.2.5 Evaluation of Mitochondrial membrane potential

JC-1 is sensitive to the Δψ and in normal mitochondria, it enters into the mitochondria forming J-aggregate and emits red fluorescence. On the other hand, in altered mitochondrial function, the dye remains in monomeric form and retains its original green fluorescence ^11,12^.

**Protocol:**

Cells were cultured in a 6-well plate at 5 x 10⁵ cells per 2ml and incubated in CO_2_ incubator overnight for 24 hours at 37°C. The spent medium was removed followed by washing with 1000μl of 1XPBS. The cells were treated with Rotenone (10uM/ml) for 24 hours to induce neurotoxicity except untreated groups. After 24 hours, desired concentrations of test agents were added in 2ml of culture medium and incubated for 24 hours. Cells without rotenone treatment are considered as an untreated group. The medium was withdrawn from all the wells and placed into 12x75 mm polystyrene tubes, which were then washed with 1000μl PBS (PBS was saved in the same tubes). PBS was withdrawn and 250μl of trypsin-EDTA solution was added followed for 3-4 minutes of incubation at 37°C. The culture media was poured back into their respective wells and the cells were harvested directly into culture 12x75mm polystyrene tubes. The tubes were centrifuged for 5 minutes at 300x g at 25°C and the supernatant was carefully decanted. JC-1 working solution of 0.5ml was added to each pellet. The cells were gently resuspended in the JC-1 working solution and vortexed to disrupt any cell-to-cell clumping followed by incubation for at 37°C in CO_2_ incubator for 10 to 15 minutes. Further the cells were washed twice. First wash was done by adding 2 ml of 1×Assay Buffer to all the tubes and cells were gently resuspended followed by vortexing to disrupt any cell-to-cell clumping. The cells were centrifuged at 400 × g for 5 min and the supernatant was carefully removed and discarded. Second washing of cells was done by adding 1ml of 1×Assay Buffer to all the tubes. The cells were gently resuspended and were vortexed to disrupt any cell-to-cell clumping. Further, centrifugation at 400×g for 5 minutes was carried out and the supernatant was carefully removed and discarded. The cell pellet was gently resuspended in 0.5 ml of 1×Assay Buffer and vortexed to disrupt any cell-to-cell clumping. The cells were acquired by flow cytometry using the FL1 and FL2 Channels. The data was analysed by BD Cell Quest Pro software.

S.2.6 Estimation of Reactive oxygen species (ROS)

Briefly, 100 ml of standard and samples were placed in the appropriate wells of an ELISA plate (ROS) and incubated for 80 minutes at 37°C. The wells were aspirated and washed three times with 0.2 ml of 1X wash buffer. After removing all of the water from the wells, 0.1 mL of Biotinylated Anitbody working solution was added to each well and incubated for 50 minutes at 37°C. After incubation, the wells were aspirated and washed three times with 1X wash buffer. Each well was filled with 0.l ml of Streptavidin-HRP working solution and incubated for 50 minutes at 37°C. After washing the wells five times, 0.09ml of TMB substrate solution was added to each well and maintained at 37°C for 30 minutes before adding 0.05ml of stop reagent to colour the solution yellow. The readings were then taken in a TECAN SPARK multi-mode plate reader at 450nm. The obtained data was analysed and plotted to produce the results.

S.2.7 Estimation of Superoxide dismutase (SOD)

Briefly, 100 mL of standard and samples were placed in the appropriate wells of an ELISA plate (SOD) and incubated for 80 minutes at 37°C. The wells were aspirated and washed three times with 0.2ml of 1Xwash buffer. After removing all of the water from the wells, 0.1 mL of Biotinylated Anitbody working solution was added to each well and incubated for 50 minutes at 37°C. After incubation, the wells were aspirated and washed three times with 1X wash buffer. Each well was filled with 0.l ml of Streptavidin-HRP working solution and incubated for 50 minutes at 37°C. After washing the wells five times, 0.09ml of TMB substrate solution was added to each well and maintained at 37°C for 30 minutes before adding 0.05ml of stop reagent to colour the solution yellow. The readings were then taken in a TECAN SPARK multi-mode plate reader at 450nm. The obtained data was analysed and plotted to produce the results.

S.2.8 Estimation of Catalase (CAT)

The standard and samples (100 ml) were placed in the appropriate wells of an ELISA plate (CAT) and incubated for 80 minutes at 37°C. The wells were aspirated and washed three times with 0.2ml of 1X wash buffer. After removing all of the water from the wells, 0.1 mL of Biotinylated Antibody working solution was added to each well and incubated for 50 minutes at 37°C. After incubation, the wells were aspirated and washed three times with 1X wash buffer. Each well was filled with 0.l ml of Streptavidin-HRP working solution and incubated for 50 minutes at 37°C. After washing the wells five times, 0.09ml of TMB substrate solution was added to each well and maintained at 37°C for 30 minutes before adding 0.05ml of stop reagent to colour the solution yellow. The readings were then taken in a TECAN SPARK multi-mode plate reader at 450nm. The obtained data was analysed and plotted to produce the results.

S.2.9 SNCA expression studies (RT-PCR)

Trizol based RNA Extraction

The cells were collected in eppendorf tubes. 1ml of TRIsoln was added to the sample and was mixed by repetitive pipetting. The homogenized sample was incubated for 5 minutes at room temperature to completely dissociate nucleoprotein complex. 0.2ml of chloroform per 1ml of TRIsoln reagent was added, mixed thoroughly and incubated at room temperature for 2-3 minutes. Centrifugation was carried out at 12000 rpm for 15 minutes at 4°C. After centrifugation, three different layers of mixture was formed which are coloured lower phenol-chloroform phase, an interphase and a colourless aqueous phase. The RNA is predominantly present in the aqueous phase, thus the aqueous phase was transferred to a fresh 1.5ml micro centrifuge tube. Then, 0.5ml of isopropanol was added for every 1ml of TRIsoln and mixed gently. The samples were incubated at room temperature for 10 minutes. Centrifugation of the samples was done at 12000 rpm for 10 minutes at 4°C. The supernatant was decanted. The RNA precipitate was observed on the side of the tube. The RNA pellet was washed once with 75% ethanol (1ml of 75% ethanol per ml of TRIsoln) after which it was centrifuged at 10000 rpm for 10 minutes at 4°C. The pellet was then kept for air-drying. The RNA pellet was resuspended in 50μl of RNase free water.

cDNA synthesis

The cDNA first strand reaction mixture was prepared by using sample, 10X RT Mix, MMLV RT enzyme and DEPC Water (up to (20μl). It was incubated at 42°C for 20min. Heat inactivated the RT reaction at 85°C for 5 min and then snap-chill the tube on ice. The first stand cDNA reaction mix was used for PCR amplification.

Primer Sequence

The following sequences were used:

Table S18. Reverse and forward sequence of SNCA

| **Name** | **Sequence** |
| --- | --- |
| SNCA F | ATCAAGACTACGAACCTGAAGC |
| SNCA R | CACATTGGAACTGAGCACTTG |

Experimental Conditions

**Primers:** The SNCA and β-actin from Sigma-Aldrich were procured and used in the experiment

PCR Reaction Mix:

1. 2X Green Mastermix for RT PCR
2. 20X Forward Primer
3. 20X Reverse Primer
4. cDNA
5. Nuclease free water (make up to 20ul)

Table S19. PCR conditions

|  | **SNCA** |
| --- | --- |
| **Initial denaturation** | 95°C (2mins) |
| **Denaturation** | 95°C (15sec) |
| **Annealing** | 56°C (30sec) |
| **Number of cycles** | 40 |
| **Channel** | FAM |

Quantification was done by using nanodrop spectrophotometer.

The delta-delta cycle threshold (Ct) method (2–∆∆Ct) was used to quantify the relative fold gene expression of all the samples. The number of cycles required for the fluorescent signal to cross the threshold is expressed in Ct. The amount of target nuclei acid in the sample is inversely proportional to the Ct level, i.e. the lower Ct represents the target nuclei in the sample.

Table S20. Collection of blood and tissue samples in staggered manner

| Animal groups | | Time in hours | | | | | | | |
| --- | --- | --- | --- | --- | --- | --- | --- | --- | --- |
|  |  | 0 | 0.5 | 1 | 3 | 5 | 7 | 24 | 48 |
| Control | 1 | B | B | N | B | N | B | N | S-T |
|  | 2 | B | B | N | B | N | B | N | B |
|  | 3 | B | B | N | B | N | S-T | - | - |
|  | 4 | B | B | N | S-T | - | - | - | - |
|  | 5 | B | S-T | - | - | - | - | - | - |
|  | 6 | B | N | S-T | - | - | - | - | - |
|  | 7 | B | N | B | N | S-T | - | - | - |
|  | 8 | B | N | B | N | B | N | S-T | - |
|  | 9 | B | N | B | N | B | N | B | S-T |
|  | 10 | B | N | B | N | B | N | S-T | - |
| Naïve-FMT  (25mg/kg, p.o.) | 1 | B | B | N | B | N | B | N | B |
|  | 2 | B | B | N | B | N | B | N | S-T |
|  | 3 | B | B | N | B | N | S-T | - | - |
|  | 4 | B | B | N | S-T | - | - | - | - |
|  | 5 | B | S-T | - | - | - | - | - | - |
|  | 6 | B | N | B | N | S-T | - | - | - |
|  | 7 | B | N | B | N | B | N | S-T | - |
|  | 8 | B | N | B | N | B | N | B | S-T |
|  | 9 | B | N | B | N | B | N | S-T | - |
|  | 10 | B | N | B | N | B | N | B | B |
| FMT-SLNs-PS80  (140mg/kg, p.o.) | 1 | B | B | N | B | N | B | N | S-T |
|  | 2 | B | B | N | B | N | B | N | B |
|  | 3 | B | B | N | B | N | S-T | - | - |
|  | 4 | B | B | N | S-T | - | - | - | - |
|  | 5 | B | S-T | - | - | - | - | - | - |
|  | 6 | B | N | S-T | - | - | - | - | - |
|  | 7 | B | N | B | N | S-T | - | - | - |
|  | 8 | B | N | B | N | B | N | S-T | - |
|  | 9 | B | N | B | N | B | N | B | S-T |
|  | 10 | B | N | B | N | B | N | S-T | - |

B: Blood, S-T: sacrificed and collected tissues, N: none

S2.10. Biochemical studies ^13^

Superoxide Dismutase (SOD*)* level

Supernatant (0.1ml), EDTA (1 × 10^−4^M, 0.1 ml), carbonate buffer (pH 9.7, 0.5 ml), and epinephrine (1 mM, 1 mL) were mixed. Spectrophotometer was used to read the optical density of formed adrenochrome read at 480nm for 3 minutes. The enzyme activity was measured in units per minute per milligrams terms (U/min/mg) of protein. Under the stated assay conditions, one unit of enzyme activity is defined as the concentration required that can inhibit the chromogen production by 50% in one minute.

Catalase (CAT) Level

The assay mixture was prepared by mixing 0.05 ml of tissue homogenate supernatant (10%) and 1.95 ml of 50mM phosphate buffer (pH 7.0) in 3 ml cuvette. 1 mL of 30mM hydrogen peroxide (H_2_O_2_) was added and variations in absorbance were measured at 240nm for 30seconds at 15 seconds intervals. The catalase activity was quantified by using the millimolar extinction coefficient of H_2_O_2_ (0.071 mmol cm^−1^) which was expressed as micromoles of H_2_O_2_ oxidised per minute per milligram protein:

CAT activity= O.D/E x sample volume (ml) x protein (mg).

Where, O.D. is the change in absorbance per minute and E is the H_2_O_2_ extinction coefficient (0.071 mmol cm^-1^)

**References:**

1 Zhang, X. *et al.* Study on pharmacokinetics and tissue distribution of single dose oral tryptanthrin in Kunming mice by validated reversed-phase high-performance liquid chromatography with ultraviolet detection. *Integrative medicine research* **6**, 269-279 (2017).

2 Deng, Z. *et al.* Pharmacokinetics and tissue distribution study of 16-dehydropregnenolone liposome in female mice after intravenous administration. *Drug delivery* **23**, 2787-2795 (2016).

3 US-FDA, F. Drug administration, FDA guidance for industry: Bioanalytical method validation, draft guidance. US Department of Health and Human Services, FDA. *Center for Drug Evaluation and Research, Rockville, MD, USA, in: https://www. fda. gov/downloads/drugs/guidances/ucm070107. Pdf* (2018).

4 Sood, S., Jawahar, N., Jain, K., Gowthamarajan, K. & Nainar Meyyanathan, S. Olanzapine loaded cationic solid lipid nanoparticles for improved oral bioavailability. *Current Nanoscience* **9**, 26-34 (2013).

5 Bhalekar, M., Upadhaya, P. & Madgulkar, A. Formulation and characterization of solid lipid nanoparticles for an anti-retroviral drug darunavir. *Applied Nanoscience* **7**, 47-57 (2017).

6 Naguib, Y. W. *et al.* Solid lipid nanoparticle formulations of docetaxel prepared with high melting point triglycerides: in vitro and in vivo evaluation. *Molecular pharmaceutics* **11**, 1239-1249 (2014).

7 Neupane, Y. R., Sabir, M., Ahmad, N., Ali, M. & Kohli, K. Lipid drug conjugate nanoparticle as a novel lipid nanocarrier for the oral delivery of decitabine: ex vivo gut permeation studies. *Nanotechnology* **24**, 415102 (2013).

8 Gerlier, D. & Thomasset, N. Use of MTT colorimetric assay to measure cell activation. *Journal of immunological methods* **94**, 57-63 (1986).

9 Mosmann, T. Rapid colorimetric assay for cellular growth and survival: application to proliferation and cytotoxicity assays. *Journal of immunological methods* **65**, 55-63 (1983).

10 Alley, M. *et al.* in *Proceedings of the American Association for Cancer Research.* 389-389 (AMER ASSOC CANCER RESEARCH PUBLIC LEDGER BLDG, SUITE 816, 150 S …).

11 Sivandzade, F., Bhalerao, A. & Cucullo, L. Analysis of the mitochondrial membrane potential using the cationic JC-1 dye as a sensitive fluorescent probe. *Bio-protocol* **9** (2019).

12 Mathur, A., Hong, Y., Kemp, B. K., Barrientos, A. A. & Erusalimsky, J. D. Evaluation of fluorescent dyes for the detection of mitochondrial membrane potential changes in cultured cardiomyocytes. *Cardiovascular Research* **46**, 126-138 (2000).

13 Bhangale, J. O. & Acharya, S. R. Anti-Parkinson activity of petroleum ether extract of Ficus religiosa (L.) leaves. *Advances in pharmacological sciences* **2016** (2016).
